# Neural Variability and Cognitive Control in Individuals With Opioid Use Disorder

**DOI:** 10.1001/jamanetworkopen.2024.55165

**Published:** 2025-01-17

**Authors:** Jean Ye, Saloni Mehta, Hannah Peterson, Ahmad Ibrahim, Gul Saeed, Sarah Linsky, Iouri Kreinin, Sui Tsang, Uzoji Nwanaji-Enwerem, Anthony Raso, Jagriti Arora, Fuyuze Tokoglu, Sarah W. Yip, C. Alice Hahn, Cheryl Lacadie, Abigail S. Greene, R. Todd Constable, Declan T. Barry, Nancy S. Redeker, H. Klar Yaggi, Dustin Scheinost

**Affiliations:** 1 Interdepartmental Neuroscience Program, Yale University, New Haven, Connecticut; 2 Department of Radiology & Biomedical Imaging, Yale School of Medicine, New Haven, Connecticut; 3 Department of Health Policy, Vanderbilt University, Nashville, Tennessee; 4 Department of Internal Medicine, Yale School of Medicine, New Haven, Connecticut; 5 Department of Internal Medicine, Roger Williams Medical Center, Providence, Rhode Island; 6 Yale School of Nursing, New Haven, Connecticut; 7 Pulmonary, Critical Care and Sleep Medicine, Yale School of Medicine, New Haven, Connecticut; 8 Program of Aging, Yale University, New Haven, Connecticut; 9 Frank H. Netter MD School of Medicine, Quinnipiac University, Hamden, Connecticut; 10 Department of Psychiatry, Yale School of Medicine, New Haven, Connecticut; 11 Child Study Center, Yale School of Medicine, New Haven, Connecticut; 12 Yale Center for Clinical Investigation, Yale School of Medicine, New Haven, Connecticut; 13 Department of Psychiatry, Brigham and Women’s Hospital, Boston, Massachusetts; 14 Department of Biomedical Engineering, Yale School of Engineering and Applied Science, New Haven, Connecticut; 15 Department of Neurosurgery, Yale School of Medicine, New Haven, Connecticut; 16 Department of Research, APT Foundation, New Haven, Connecticut; 17 School of Nursing, University of Connecticut, Storrs; 18 Clinical Epidemiology Research Center, VA CT Healthcare System, West Haven, Connecticut; 19 Department of Statistics & Data Science, Yale School of Medicine, New Haven, Connecticut

## Abstract

**Importance:**

Opioid use disorder (OUD) impacts millions of people worldwide. Prior studies investigating its underpinning neural mechanisms have not often considered how brain signals evolve over time, so it remains unclear whether brain dynamics are altered in OUD and have subsequent behavioral implications.

**Objective:**

To characterize brain dynamic alterations and their association with cognitive control in individuals with OUD.

**Design, Setting, and Participants:**

This case-control study collected functional magnetic resonance imaging (fMRI) data from individuals with OUD and healthy control (HC) participants. The study was performed at an academic research center and an outpatient clinic from August 2019 to May 2024.

**Exposure:**

Individuals with OUD were all recently stabilized on medications for OUD (<24 weeks).

**Main Outcomes and Measures:**

Recurring brain states supporting different cognitive processes were first identified in an independent sample with 390 participants. A multivariate computational framework extended these brain states to the current dataset to assess their moment-to-moment engagement within each individual. Resting-state and naturalistic fMRI investigated whether brain dynamic alterations were consistently observed in OUD. Using a drug cue paradigm in participants with OUD, the association between cognitive control and brain dynamics during exposure to opioid-related information was studied. Variations in continuous brain state engagement (ie, state engagement variability [SEV]) were extracted during resting-state, naturalistic, and drug-cue paradigms. Stroop assessed cognitive control.

**Results:**

Overall, 99 HC participants (54 [54.5%] female; mean [SD] age, 31.71 [12.16] years) and 76 individuals with OUD (31 [40.8%] female; mean [SD] age, 39.37 [10.47] years) were included. Compared with HC participants, individuals with OUD demonstrated consistent SEV alterations during resting-state (99 HC participants; 71 individuals with OUD; *F*_4,161_ = 6.83; *P* < .001) and naturalistic (96 HC participants; 76 individuals with OUD; *F*_4,163_ = 9.93; *P* < .001) fMRI. Decreased cognitive control was associated with lower SEV during the rest period of a drug cue paradigm among 70 participants with OUD. For example, lower incongruent accuracy scores were associated with decreased transition SEV (ρ_58_ = 0.34; *P* = .008).

**Conclusions and Relevance:**

In this case-control study of brain dynamics in OUD, individuals with OUD experienced greater difficulty in effectively engaging various brain states to meet changing demands. Decreased cognitive control during the rest period of a drug cue paradigm suggests that these individuals had an impaired ability to disengage from opioid-related information. The current study introduces novel information that may serve as groundwork to strengthen cognitive control and reduce opioid-related preoccupation in OUD.

## Key Points

**Question:**

Do individuals with opioid use disorder (OUD) engage recurring brain states differently, and are such brain dynamic alterations associated with worse cognitive control among individuals with OUD?

**Findings:**

In this case-control study, individuals recently stabilized on medications for OUD demonstrated consistent brain dynamic alterations compared with healthy control participants during rest and movie-watching. In individuals with OUD, worse cognitive control was associated with decreased flexibility at the neural level when individuals were exposed to opioid-related stimuli.

**Meaning:**

These findings suggest that future interventions targeting brain dynamic alterations in OUD may support individuals to flexibly disengage from opioid-related information to decrease preoccupation as well as substance-seeking behaviors.

## Teaser

This case-control study compares functional magnetic resonance imaging data from individuals with opioid use disorder (OUD) and healthy control participants to examine whether individuals with OUD have different brain dynamic patterns than their peers.

## Introduction

Opioid use disorder (OUD) is a chronic condition impacting millions of people globally.^1^ Individuals with OUD face increased risks of injuries, incarceration, and premature mortality.^2,3^ One prominent characteristic of OUD is altered cognitive control.^4,5^ Individuals with OUD showed worse performance when the need to suppress automatic response arose.^6,7,8^ This was accompanied by hypoactivation in the anterior cingulate and the lateral and medial prefrontal cortex.^8,9,10^ Elucidating the etiology of altered cognitive control in OUD may lead to more targeted interventions.

While neuroimaging studies have greatly expanded our understanding of OUD and its related symptoms,^9,11,12^ they adopt a static approach where brain activity is averaged over a minutes-long scan. Thus, this method cannot track how brain activity changes over time when such dynamics may better characterize symptoms associated with OUD. Specifically, variation in brain signals over time (ie, neural variability) supports flexible responses to evolving demands.^13,14,15^ The inability to flexibly disengage from opioid-related information may relate to worse cognitive control and preoccupation, an important stage in the addiction cycle.^16^ Therefore, investigating neural variability may lead to new insights about OUD.

Examining brain dynamics often involves the identification of brain states (ie, recurring brain activity patterns). How much time individuals spent in or how frequently they transitioned between brain states have been linked to psychiatric symptoms, such as hallucination, rumination, and dependence severity.^17,18,19,20^ Recent work has begun to apply brain states to understand dynamic alterations in OUD.^21^ However, it remains unclear what behavioral implications altered brain dynamics may have in OUD. Additionally, despite the growing popularity of studying brain states, previous work has not examined variability in the moment-to-moment engagement of a given brain state (ie, state engagement variability [SEV]). This measure may be promising, as it combines both brain states’ potential to capture clinically relevant information and neural variability’s relevance for cognitive control.

To address this gap, we leveraged a new multivariate computational framework to perform an exploratory study on SEV in OUD. By allowing brain states to overlap temporally and spatially, this framework tracks multiple brain states continuously at the resolution of individual time points. We focused specifically on individuals with OUD who recently stabilized on medication for OUD (MOUD). We investigated whether SEV may be altered in OUD using naturalistic functional magnetic resonance imaging (fMRI), which presents an opportunity to investigate brain dynamic alterations in a relatively ecologically valid and undisrupted manner.^22,23^ Previous work noted worse cognitive control in individuals with OUD. We hypothesized that they would show lower SEV than healthy control (HC) participants. Supplementary analysis was performed with resting-state fMRI to explore altered SEV as a consistent characteristic of OUD. Next, we investigated whether neural variability in OUD was associated with cognitive control. We focused specifically on the resting period during a drug cue paradigm when no opioid-related information was presented. We hypothesized that lower cognitive control would be associated with decreased SEV during this rest period, indicating reduced flexibility at the neural level when there was an opportunity to disengage from opioid-related information.

## Methods

### Participants

Data from 2 datasets were analyzed. The Yale University institutional review board and the Yale MRRC Protocol Review Committee approved both studies. Informed consent was obtained from all participants. Self-reported demographic characteristics, including sex and race, were collected from participants. Race data were collected to gain an understanding of the demographic characteristics of the participants. HC participants and individuals with OUD were recruited through 2 different studies with different response options for race. In the dataset with individuals with OUD, participants who did not report race information were included as unknown. In the study recruiting healthy controls, participants were given an additional option of other where they could specify their own response. One dataset included individuals who met *Diagnostic and Statistical Manual of Mental Disorders* (Fifth Edition) (*DSM-5*) criteria for OUD and were stabilized on MOUD (<24 weeks). Naturalistic, resting-state, and drug cue fMRI data were collected from these individuals. During the 6-minute naturalistic paradigm, participants watched 3 movie clips presented in the same order without a break (*Inside Out*, *The Princess Bride*, and *Up)*. We additionally collected a 6-minute eyes-open, resting-state scan. In the drug cue paradigm, participants were presented with either a rest block (a white crosshair presented in the center of a black screen) or a cue block (a picture showing opioid-related stimuli; eg, a needle or a bottle of pills). There were 9 alternating rest and cue blocks, each lasting 16 seconds. To distinguish from resting-state fMRI, we will refer to the rest in the drug cue paradigm as the rest period or condition. While all individuals with OUD participated in neuroimaging, not all completed or had usable data for all 3 conditions. To retain as many participants as possible for each analysis, we performed exclusion for each condition separately (eAppendix in Supplement 1). We also included HC participants from a separate transdiagnostic dataset. Participants in this dataset were only included if they had no neurological or mental health diagnoses based on self-report and the Mini-International Neuropsychiatric Interview for *DSM-5*. Naturalistic and resting-state fMRI data were collected using the same paradigm described previously. No drug cue data were collected in this dataset. This report adheres to the Strengthening the Reporting of Observational Studies in Epidemiology (STROBE) guidelines.

### fMRI Data Preprocessing

Detailed acquisition and preprocessing information can be found in the eAppendix in Supplement 1. A standard preprocessing pipeline^24^ was applied to the structural and functional data. A combination of nonlinear and linear transformations aligned the functional data to the template space before time series data was parcellated with the Shen-268 atlas.

Additional quality control included removing participants with mean framewise displacement over 0.2 mm, missing time points, or brain coverage. After these criteria, 96 HC participants and 76 individuals with OUD were included in the naturalistic fMRI analysis; resting-state data from 99 HC participants and 71 individuals with OUD were retained (exclusion criteria detailed in the eAppendix in Supplement 1). We analyzed the drug cue fMRI data from 70 individuals with OUD.

### Brain Dynamic Measures

**Figure 1.**
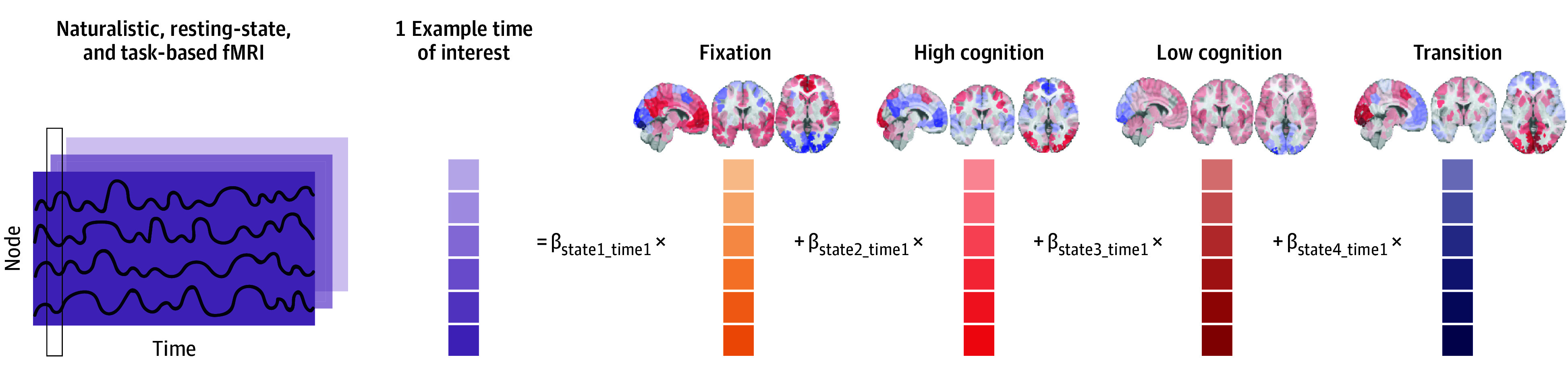
Study Pipeline Several datasets were used in this study (eFigure 1 in Supplement 1). First, 4 recurring brain states were identified using task-based functional magnetic resonance imaging (fMRI) from the Human Connectome Project dataset. Based on the prominent task conditions associated with each brain state, we characterized them as fixation, high cognition, low cognition, and transition (eTable 1 in Supplement 1). For example, the fixation brain state mainly includes time points from the fixation condition across task paradigms. In contrast, the low-cognition brain state consists mostly of time points from the motor task (eTable 1 in Supplement 1). Moment-to-moment state engagement was assessed in naturalistic, resting-state, and drug cue fMRI using nonnegative least squares regression. For a given time point, the framework can determine multiple brain states’ concurrent engagement (indicated by the β coefficients). The figure illustrates this process for 1 example time point of interest. After engagement values are extracted from all time points, variability is computed as the standard deviation of moment-to-moment engagement across time.

We leveraged a new multivariate computational framework to assess brain dynamics at the whole-brain level^24^ (eAppendix in Supplement 1; Figure 1). Following methods established by previous work (eAppendix in Supplement 1), 4 recurring brain states were identified by applying nonlinear manifold learning to task-based fMRI data from the Human Connectome Project (HCP) dataset.^25^ These brain states were characterized as fixation, high cognition, low cognition, and transition based on their associated task conditions. For instance, the fixation brain state includes time points where a crosshair was presented at the center of the screen (eTable 1 in Supplement 1). The rich HCP dataset allowed us to identify brain states underlying various cognitive processes while circumventing circular analysis. While HCP consists of healthy individuals, previous work and our preliminary analysis have revealed that similar brain states can be identified in individuals with psychopathology (eAppendix in Supplement 1).^19,26,27^

Next, our framework applied nonnegative least squares regression to extend and evaluate each state’s engagement at each time point in a different dataset. Our approach permits temporal overlap to track multiple brain states continuously. SEV was computed for each individual and each brain state by evaluating the standard deviation of moment-to-moment engagement across time (Figure 1). While this article focuses on neural variability, we also examined the amount of brain state engagement in eFigure 5 and eTables 6 and 7 in Supplement 1.

Variability was calculated across the entire run for naturalistic and resting-state fMRI. Prior work has revealed significantly higher SEV during rest compared with task,^24^ aligning with the understanding that rest is less constrained than task. Thus, we separately computed SEV within the rest and cue condition for the drug cue paradigm. A validation analysis was performed to replicate previous results. Higher SEV during rest than cue condition indicates that participants may be more flexible at the neural level when not constrained by task. Along the same lines, lower SEV during the rest condition may suggest the constraint from task has spread into a period where individuals could flexibly disengage from opioid-related information.

### Stroop-Assessed Cognitive Control

Individuals with OUD additionally completed an out-of-scanner Stroop task. We used this task to assess cognitive control and explored the behavioral implications of SEV alterations. While the Stroop interference score is a popular measure to assess cognitive control, recent literature has noted concerns regarding the difference score’s test-retest reliability, convergent validity, and criterion validity.^28,29,30,31,32,33^ Given the performance score from a single condition may be a more robust indicator of cognitive control,^29,34,35,36,37^ we focused on the accuracy (ACC) and accurate trial mean response time (RT) from the incongruent condition. Individuals with missing or outlier behavioral scores were excluded. We included 60 and 57 participants with OUD in the final analysis for ACC and RT analysis, respectively (eAppendix in Supplement 1).

### Statistical Analysis

Multivariate group differences in SEV were examined with Hotelling *T*^2^ test. Age and sex were included as covariates. Sex × group and age × group interactions were explored. We correlated Stroop performance with SEV during the drug cue paradigm using Spearman correlation. The first rest block was excluded for consistency since no prior stimuli were presented. We examined SEV extracted from both conditions for completeness. Results for these analyses were false discovery rate–corrected for multiple comparisons. We consider *P* < .05 significant. Analyses were conducted with R version 4.3.1 (R Project for Statistical Computing) and MATLAB version R2021a (MathWorks).

## Results

### Demographic Data

Demographic information for HC participants and individuals with OUD is included in Table 1. All individuals with OUD were stabilized on methadone and met *DSM-5* criteria for either moderate or severe OUD (eTable 3 in Supplement 1). HC participants were significantly younger than individuals with OUD (movie: 96 HC participants, mean [SD] age, 31.77 [12.19] years; 76 individuals with OUD, mean [SD] age, 39.37 [10.47] years; *t*_168.85_ = −4.39, *P* < .001; resting-state: 99 HC participants, mean [SD] age, 31.71 [12.16] years; 71 individuals with OUD, mean [SD] age, 39.79 [10.97] years; *t*_159.31_ = −4.53, *P* < .001), but the 2 groups did not differ in sex (movie: 53 female HC participants [55.2%]; 31 female individuals with OUD [40.8%]; χ^2^ = 2.98; *P* = .09; resting-state: 54 female HC participants [54.5%]; 30 female individuals with OUD [42.3%]; χ^2^ = 2.03; *P* = .15).

**Table 1.** Demographic Information for Both Datasets

Characteristic	Participants, No. (%)
**Naturalistic paradigm, healthy control participants (n = 96)**	
Self-reported sex assigned at birth	
Female	53 (55.2)
Male	43 (44.8)
Age, mean (SD), y	31.77 (12.19)
Self-reported race^a^	
Asian or Pacific Islander	17 (17.7)^b^
Biracial (Hispanic)	1 (1.0)
Biracial (not of Hispanic origin)	2 (2.1)
Black (Hispanic)	2 (2.1)
Black (not of Hispanic origin)	21 (21.9)
Native American	1 (1.0)^b^
Other (specify)	1 (1.0)
White (Hispanic)	9 (9.4)
White (not of Hispanic origin)	44 (45.8)
**Naturalistic paradigm, individuals with OUD (n = 76)**	
Self-reported sex assigned at birth	
Female	31 (40.8)
Male	45 (59.2)
Age, mean (SD), y	39.37 (10.47)
Self-reported race	
American Indian or Alaska Native	0
Asian	0
Black or African American	2 (2.6)
More than 1 race	6 (7.9)
Native Hawaiian or Other Pacific Islander	0
Unknown	3 (3.9)
White	65 (85.5)
OUD duration, mean (SD), y^c^	14.82 (9.93)
OUD severity^d^	
Moderate	4 (5.3)
Severe	72 (94.7)
Methadone dose at baseline, mean (SD), mg	81.95 (22.64)
**Resting-state, healthy control participants (n = 99)**	
Self-reported sex assigned at birth	
Female	54 (54.5)
Male	45 (45.5)
Age, mean (SD), y	31.71 (12.16)
Self-reported race^a^	
Asian or Pacific Islander	18 (18.2)^e^
Biracial (Hispanic)	2 (2.0)
Biracial (not of Hispanic origin)	2 (2.0)
Black (Hispanic)	2 (2.0)
Black (not of Hispanic origin)	21 (21.2)
Native American	1 (1.0)^b^
Other (specify)	1 (1.0)
White (Hispanic)	9 (9.1)
White (not of Hispanic origin)	46 (46.5)
**Resting-state, individuals with OUD (n = 71)**	
Self-reported sex assigned at birth	
Female	30 (42.3)
Male	41 (57.7)
Age, mean (SD), y	39.79 (10.97)
Self-reported race^a^	
American Indian or Alaska Native	0
Asian	0
Black or African American	2 (2.8)
More than 1 race	7 (9.9)
Native Hawaiian or other Pacific Islander	0
Unknown	4 (5.6)
White	58 (81.7)
OUD duration, mean (SD), y^c^	15.07 (10.67)
OUD severity^d^	
Moderate	2 (2.8)
Severe	69 (97.2)
Methadone dose at baseline, mean (SD), mg	81.83 (22.59)
**Drug cue paradigm, individuals with OUD (n = 70)**	
Self-reported sex assigned at birth	
Female	31 (44.3)
Male	39 (55.7)
Age, mean (SD), y	39.20 (10.69)
Self-reported race^a^	
American Indian or Alaska Native	0
Asian	0
Black or African American	2 (2.9)
More than 1 race	6 (8.6)
Native Hawaiian or other Pacific Islander	0
Unknown	3 (4.3)
White	59 (84.3)
OUD duration, mean (SD), y^c^	14.69 (9.91)
OUD severity^d^	
Moderate	3 (4.3)
Severe	67 (95.7)
Methadone dose at baseline, mean (SD), mg	81.50 (22.85)

Abbreviation: OUD, opioid use disorder.

^a^ Race information was self-reported. Healthy control participants and individuals with OUD were recruited through 2 different studies with different response options for race. The complete list of options for each study is listed in the table. In the dataset with individuals with OUD, participants who did not report race information were included as unknown. In the study recruiting healthy controls, participants were given an additional option of other where they could specify their own response. The participant who checked other did not provide additional information. No further instructions were given to participants when they filled out the form.

^b^ One participant from these groups also selected White (not Hispanic).

^c^ OUD duration was computed as the difference between participant’s current and the age of *Diagnostic and Statistical Manual of Mental Disorders* (Fifth Edition) OUD symptom onset.

^d^ OUD severity was measured using the Structured Clinical Interview for *DSM-5* disorders, based on *Diagnostic and Statistical Manual of Mental Disorders* (Fifth Edition) (mild, 2-3 symptoms; moderate, 4-5 symptoms; severe, 6 or more symptoms).

^e^ Two participants from this group also selected White (not Hispanic).

### SEV in Individuals With OUD

**Figure 2.**
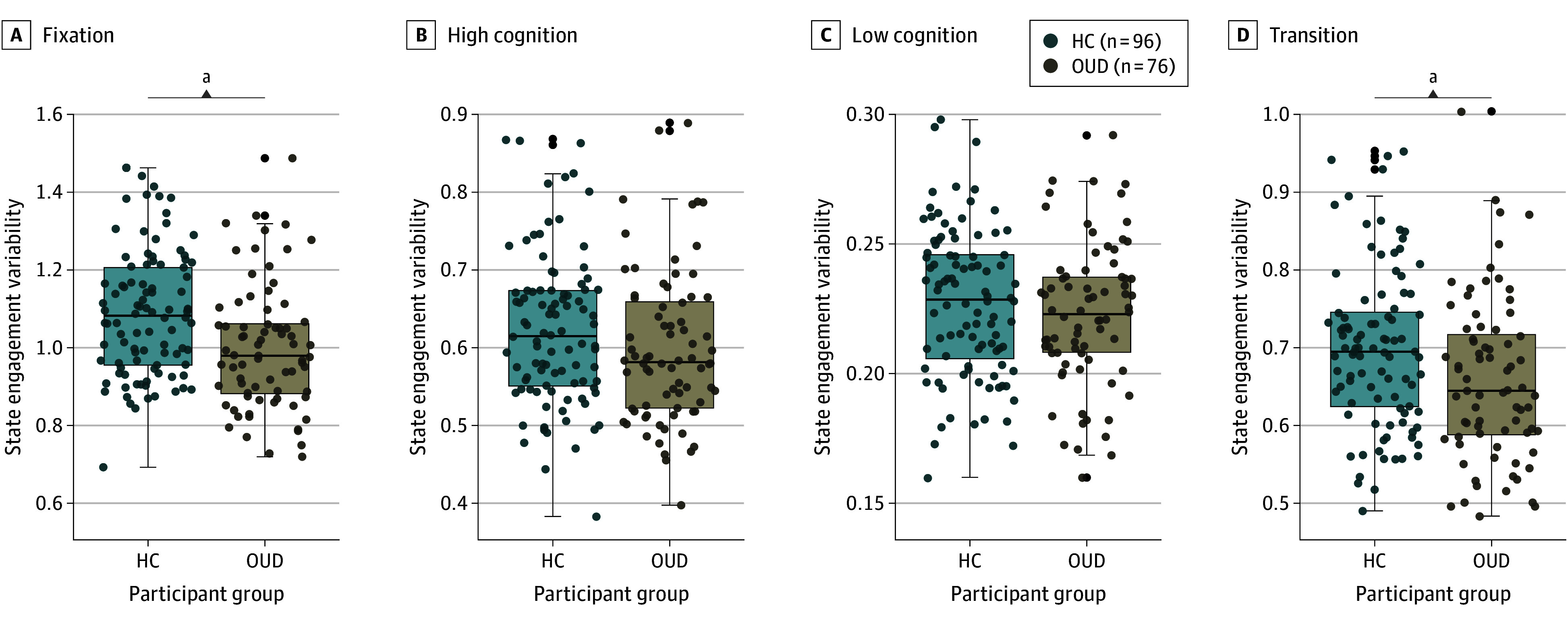
Group Differences During Movie-Watching In each panel, the box edges indicate the interquartile range. The center line shows the median. The whiskers extend to show the minimum and maximum values. Each dot represents data from a participant. Black dots indicate participants with outlier data. Individuals with opioid use disorder (OUD) had lower fixation and transition state engagement variability than healthy control (HC) participants. ^a^*P* < .05 when each state was examined individually in post hoc analyses of variance.

During movie-watching, we observed a significant association between group and SEV (*F*_[4,163]_ = 9.93; *P* < .001) (Figure 2A and eFigure 3A in Supplement 1). The median (IQR) SEV for fixation for HC participants was 1.08 (0.96-1.21); it was 0.98 (0.88-1.06) for participants with OUD. For transition, the median (IQR) was 0.69 (0.62-0.75) for HC participants and 0.64 (0.59-0.72) for participants with OUD. There was no sex association (*F*_4,163_ = 1.44; *P* = .22) or sex × group interaction (*F*_4,163_ = 0.31; *P* = .87). Consistent with previous work,^24^ age showed a significant association with SEV (*F*_4,163_ = 6.53; *P* < .001). There was no age × group interaction (*F*_4,164_ = 2.26; *P* = .07).

Post hoc analyses of variance interrogated group differences in each brain state separately (eTable 4 in Supplement 1). Fixation and transition SEV were significantly lower in individuals with OUD compared with HC participants (mean [SD] fixation, HC: 1.09 [0.16]; OUD: 1.00 [0.16]; *F*_1,166_ = 12.74; *P* < .001; mean [SD] transition, HC: 0.70 [0.10]; OUD, 0.66 [0.10]; *F*_1,166_ = 7.06; *P* = .009). High cognition (*F*_1,166_ = 2.62; *P* = .11) and low cognition (*F*_1,166_ = 1.21; *P* = .27) SEVs did not differ between groups. However, these 2 states showed significantly different levels of engagement between groups (eFigure 5 in Supplement 1). Compared with HCs, individuals with OUD engaged the low-cognition brain state more and the high-cognition brain state less (eFigure 5 in Supplement 1).

Our supplementary analysis revealed that SEV during resting-state fMRI also differed significantly between groups (*F*_4,161_ = 6.83; *P* < .001) (eFigure 3B in Supplement 1). However, results were less robust when each brain state was examined separately (eTable 5 and eFigure 4 in Supplement 1). These findings suggest that altered SEV may be a consistent characteristic of OUD, with naturalistic fMRI being more powerful at capturing such differences than rest.

### Cognitive Control and SEV During a Drug Cue Paradigm

We next investigated the behavioral implications of altered SEV using drug cue fMRI. As expected, SEV was lower during cue compared with rest (fixation: t_69_ = 2.01; *P* = .048; high cognition: t_69_ = 2.10; *P* = .04; low cognition: t_69_ = 4.62; *P* < .001; transition: t_69_ = 2.14; *P* = .04). These patterns remained consistent when we introduced lags to task time indices (eTable 8 in Supplement 1). This validation analysis suggests that the brain may be more constrained during cue than rest, showing that our measures are sensitive to how the brain flexibly responds to changing demands in the environment.

**Figure 3.**
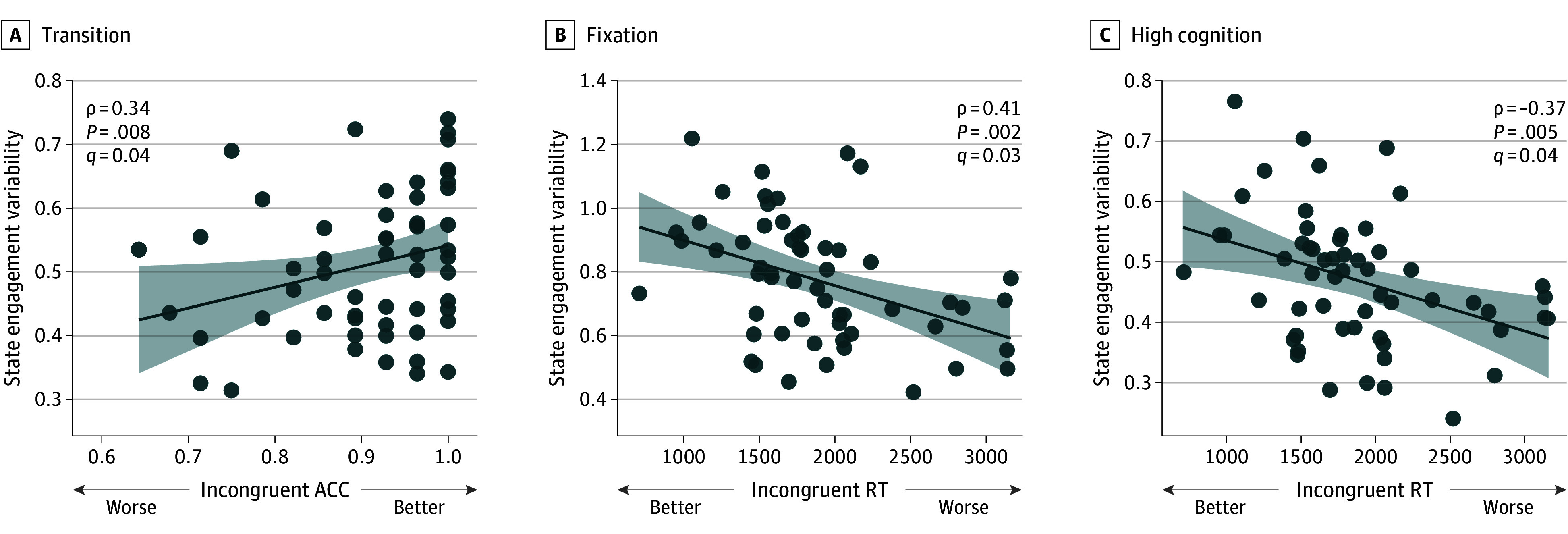
State Engagement Variability and Behavioral Performance During the Incongruent Condition During the rest condition of the drug cue paradigm, worse accuracy (ACC) was associated with lower transition state engagement variability. Worse response time (RT) performance was associated with lower fixation and high-cognition state engagement variability. Each dot represents a participant. The line represents a linear fit to the data, and the shading area represents the 95% CIs for this linear model.

The association between cognitive control and SEV was examined. Lower incongruent ACC score was associated with decreased transition SEV (ρ_58_ = 0.34; *P* = .008; *q* = .04). Worse incongruent RT was associated with lower fixation (ρ_55_ = −0.41; *P* = .002; *q* = .03) and lower high cognition (ρ_55_ = −0.37; *P* = .005; *q* = .04) (Figure 3 and Table 2). We additionally observed consistent results demonstrating lower SEV was associated with worse Stroop performance when cognitive control was assessed with the interference scores (eTable 9, eFigure 2, and eFigure 9 in Supplement 1).

**Table 2.** State Engagement Variability During the Drug Cue Paradigm and Cognitive Control as Assessed by Performance During Stroop Incongruent Condition

Brain state	Incongruent ACC			Incongruent RT		
	ρ	*P* value	*q*	ρ	*P* value	*q*
**State engagement variability during cue and cognitive control**						
Fixation	−0.02	.88	.95	0.06	.64	.95
High cognition	−0.01	.93	.95	−0.01	.95	.95
Low cognition	−0.02	.90	.95	0.05	.74	.95
Transition	0.04	.78	.95	0.09	.49	.87
**State engagement variability during rest condition and cognitive control**						
Fixation	0.24	.06	.20	−0.41	.002	.03
High cognition	0.22	.10	.26	−0.37	.005	.04
Low cognition	0.12	.37	.75	−0.13	.32	.74
Transition	0.34	.01	.04	−0.32	.02	.06

Abbreviations: ACC, accuracy; RT, response time.

Cognitive control measures were not associated with SEV during the cue condition. These associations remained consistent when lags were added to task time indices (eTables 10 and 11 in Supplement 1). Our results suggest that the rest period of the drug cue paradigm may contain unique insights about cognitive control. We explored the association between cognitive control and SEV extracted during resting-state fMRI as a control analysis. Notably, no significant association was found (eTable 12 in Supplement 1).

## Discussion

Using naturalistic, resting-state, and task-based fMRI, we leveraged a new multivariate computational framework to investigate brain dynamic alterations in individuals with OUD who recently stabilized on MOUD. The novelty of the current study is two-fold. First, our results suggest that altered neural variability may be a consistent characteristic of OUD. During movie-watching, individuals with OUD demonstrated lower SEV compared with HC participants. Such decreased variability may reflect impaired flexibility to engage different brain states to address evolving demands. Second, lower SEV may have behavioral relevance. Worse cognitive control was associated with decreased SEV during the rest period of a drug cue paradigm. By analyzing brain activity across time, we found that a task classically used to study one hallmark characteristic of OUD (ie, aberrant response to opioid-related stimuli) could provide information on another symptom (ie, impaired cognitive control). A traditional brain activation analysis (eFigure 6 and eTable 13 in Supplement 1) did not reveal an association between cognitive control and brain responses during the drug cue paradigm.

Altered neural variability may reflect disrupted brain network communications in OUD. This interpretation aligns with the reduced structural and functional connectivity found in mice and humans dependent on opioids.^38,39^ During movie-watching, we observed significantly reduced fixation and transition SEV in OUD. Lower SEV may suggest individuals with OUD experience lower flexibility in engaging these 2 brain states. The fixation brain state predominantly recruits the default mode network (eTable 2 in Supplement 1), which is relevant for relapse risk and withdrawal symptoms in individuals using heroin.^40,41^ The transition brain state captures activation patterns associated with a change in task demand (eTable 1 in Supplement 1). Given what brain networks are involved in these brain states, future hypothesis-driven research should investigate whether more rigid brain state engagement may contribute to greater difficulty in switching away from self-related thoughts revolving around the urge for opioid use.^16^

Consistent with prior literature suggesting that neural variability supports behavioral performance,^14,42,43^ we found that brain states showing significant group differences in SEV also demonstrated associations with cognitive control. Higher transition SEV was associated with better Stroop incongruent ACC. This result suggests that the flexible recruitment of a brain state sensitive to task demand change supports cognitive control. Furthermore, worse Stroop incongruent RT was associated with decreased fixation and high-cognition SEVs. These 2 brain states involve the deactivation and activation of brain networks implicated in high-level processing, such as the frontoparietal and default mode networks (eTable 2 in Supplement 1). Given these networks’ role in coordinating large-scale brain responses to support cognitive control,^44,45^ the flexible engagement of these brain states may allow individuals to respond during Stroop effectively.

Interestingly, significant associations between cognitive control and SEV were found in the rest but not cue condition of a drug cue paradigm. This finding dovetails with previous work suggesting that—in addition to the initial response to salient stimuli (such as opioid-related images)—how individuals recover or disengage from such stimuli conveys important information.^46,47,48^ Drug cue paradigms are a popular approach to investigate the neural mechanisms underpinning OUD.^9^ Brain responses to opioid-related stimuli are related to treatment adherence and withdrawal symptoms.^49,50^ However, less work has been focused on the rest period, with no cue presentation. Our results show that this period may serve as a novel window into studying cognitive control and the lingering of salient information in the brain. The lack of association between cognitive control and resting-state SEV provided further evidence. Unlike a regular resting-state scan, the rest period of a drug cue paradigm was surrounded by presentations of opioid-related images. This may create a unique scenario in which opioid-related processing can linger beyond the cue condition. Individuals who showed lower cognitive control also demonstrated decreased flexibility at the neural level, potentially reflecting a greater difficulty in disengaging from opioid-related information during the rest period.

However, lingering opioid-related processing is only one of many interpretations. Biased attention toward opioid-related stimuli may also constrain thoughts and reduce variability during the rest period. Both response inhibition and biased attention for substance-related stimuli are hallmark characteristics of OUD.^9,12,51,52,53^ Since we did not assess craving or perform thought sampling, understanding what contributes to lower variability during the rest period remains a future research direction.

### Limitations

The current study has several limitations. While we developed our hypothesis based on previous neural variability studies^13,14,15^ and prior observations of lower SEV in individuals with other clinical conditions,^24^ we did not preregister our analysis. This study remained exploratory. Future hypothesis-driven work should further validate our results and investigate the neural mechanisms underpinning OUD. Our brain states were identified in HCP participants. But we observed consistent results when brain states were instead identified in a patient population (eTables 14 and 15 and eFigure 7 in Supplement 1). Future studies should investigate whether the brain dynamic alterations reported here may be observed with other brain states. As no control group was included in the drug cue analysis, it remains elusive whether the association we found is unique to individuals with OUD. Further, abstinence time, treatment time, and time since the last dose can all influence brain responses to drug-related cues and cognitive control in individuals with OUD.^50,54,55^ Future work should explore the interaction between different time scales. Would individuals abstinent for longer (ie, relatively longer time scale) show different associations between behaviors and brain dynamics alterations (ie, relatively shorter time scale)? Individuals with OUD present a heterogeneous sample, with remarkable interindividual variations in risk, susceptibility, disorder profile, and progression. More understanding of how comorbidities or polysubstance use may interact with opioid use to influence brain dynamics is needed. As all participants in this study were stabilized on methadone, future investigations should also explore whether brain dynamics may be altered in similar ways across individuals with OUD taking different medications.

## Conclusions

In this case-control study of brain dynamics in OUD, we applied a multivariate framework to naturalistic, resting-state, and task-based fMRI data to examine altered brain dynamics in individuals with OUD. The observed challenges individuals with OUD have in engaging brain networks flexibly over time can have implications for cognitive control, which may be particularly relevant during exposure to opioid-related stimuli.

## Notes

Supplement 1. **eAppendix.** Supplementary Methods and Results **eFigure 1.** Study Overview **eFigure 2.** Distribution of Behavioral Performance During Stroop Incongruent Condition **eTable 1.** Number of Volumes Associated With Each Task Condition for the 4 Recurring Brain States **eTable 2.** Networks Showing the Highest Activation and Deactivation Percentages for Each State **eTable 3.** Individuals With Opioid Use Disorder Who Reported Polysubstance Use **eTable 4**. State Engagement Variability During Naturalistic Stimuli ANOVA Covariates **eFigure 3.** Distribution of State Engagement Variability During Movie Watching and Resting-State **eTable 5**. State Engagement Variability During Resting-State fMRI (ANOVAs) **eFigure 4. **Age-by-Group Interaction in State Engagement Variability During Resting-State fMRI **eFigure 5**. Group Differences in Relative State Engagement During Movie-Watching **eTable 6**. Relative State Engagement During Resting-State fMRI (ANOVAs) **eTable 7**. Relative State Engagement During Movie-Watching fMRI (ANOVAs) **eTable 8**. State Engagement Variability Between Rest and Cue Conditions **eTable 9.** State Engagement Variability During the Drug Cue Paradigm and Cognitive Control Assessed With Interference Scores **eTable 10**. Cognitive Control and State Engagement Variability During Drug Cue Paradigm (Rest Condition) With Lags Added to Task Time Indices **eTable 11**. Cognitive Control and State Engagement Variability During Drug Cue Paradigm (Cue Condition) With Lags Added to Task Time Indices **eTable 12**. Associations Between Cognitive Control and Resting-State State Engagement Variability **eFigure 6.** (A) State Engagement Variability and Stroop Interference Scores (B) Activations During the Drug Cue Task **eTable 13**. Task Activation During the Cue Paradigm and Cognitive Control **eTable 14**. Similarities Between HCP and CNP Brain States **eTable 15**. State Engagement Variability During Naturalistic fMRI Using CNP Brain States (ANOVAs) **eFigure 7.** Group Comparison Using State Engagement Variability Extracted Using CNP Brain States
eReferences.

Supplement 2.
Data Sharing Statement
