# Supplementary material for: Neural Variability and Cognitive Control in Individuals With Opioid Use Disorder
Source: JAMA Netw Open. 2025 Jan 17;8(1):e2455165. doi: 10.1001/jamanetworkopen.2024.55165 (PMC11742521; doi:10.1001/jamanetworkopen.2024.55165)
Supplement: Supplement 1. — eAppendix. Supplementary Methods and Results eFigure 1. Study Overview eFigure 2. Distribution of Behavioral Performance During Stroop Incongruent Condition eTable 1. Number of Volumes Associated With Each Task Condition for the 4 Recurring Brain States eTable 2. Networks Showing the Highest Activation and Deactivation Percentages for Each State eTable 3. Individuals With Opioid Use Disorder Who Reported Polysubstance Use eTable 4. State Engagement Variability During Naturalistic Stimuli ANOVA Covariates eFigure 3. Distribution of State Engagement Variability During Movie Watching and Resting-State eTable 5. State Engagement Variability During Resting-State fMRI (ANOVAs) eFigure 4. Age-by-Group Interaction in State Engagement Variability During Resting-State fMRI eFigure 5. Group Differences in Relative State Engagement During Movie-Watching eTable 6. Relative State Engagement During Resting-State fMRI (ANOVAs) eTable 7. Relative State Engagement During Movie-Watching fMRI (ANOVAs) eTable 8. State Engagement Variability Between Rest and Cue Conditions eTable 9. State Engagement Variability During the Drug Cue Paradigm and Cognitive Control Assessed With Interference Scores eTable 10. Cognitive Control and State Engagement Variability During Drug Cue Paradigm (Rest Condition) With Lags Added to Task Time Indices eTable 11. Cognitive Control and State Engagement Variability During Drug Cue Paradigm (Cue Condition) With Lags Added to Task Time Indices eTable 12. Associations Between Cognitive Control and Resting-State State Engagement Variability eFigure 6. (A) State Engagement Variability and Stroop Interference Scores (B) Activations During the Drug Cue Task eTable 13. Task Activation During the Cue Paradigm and Cognitive Control eTable 14. Similarities Between HCP and CNP Brain States eTable 15. State Engagement Variability During Naturalistic fMRI Using CNP Brain States (ANOVAs) eFigure 7. Group Comparison Using State Engagement Variability Extracted Using CNP Brai [file jamanetwopen-e2455165-s001.pdf]

Supplemental Online Content

Ye J, Mehta S, Peterson H, et al. Neural variability and cognitive control in individuals with opioid use disorder. *JAMA Netw Open.* 2025;8(1):e2455165. doi:10.1001/jamanetworkopen.2024.55165

eAppendix. Supplementary Methods and Results

eFigure 1. Study Overview

eFigure 2. Distribution of Behavioral Performance During Stroop Incongruent Condition

eTable 1. Number of Volumes Associated With Each Task Condition for the 4 Recurring Brain States

eTable 2. Networks Showing the Highest Activation and Deactivation Percentages for Each State

eTable 3. Individuals With Opioid Use Disorder Who Reported Polysubstance Use

eTable 4. State Engagement Variability During Naturalistic Stimuli ANOVA Covariates

eFigure 3. Distribution of State Engagement Variability During Movie Watching and Resting-State

eTable 5. State Engagement Variability During Resting-State fMRI (ANOVAs)

eFigure 4. Age-by-Group Interaction in State Engagement Variability During Resting-State fMRI

eFigure 5. Group Differences in Relative State Engagement During Movie-Watching

eTable 6. Relative State Engagement During Resting-State fMRI (ANOVAs)

eTable 7. Relative State Engagement During Movie-Watching fMRI (ANOVAs)

eTable 8. State Engagement Variability Between Rest and Cue Conditions

eTable 9. State Engagement Variability During the Drug Cue Paradigm and Cognitive Control Assessed With Interference Scores

eTable 10. Cognitive Control and State Engagement Variability During Drug Cue Paradigm (Rest Condition) With Lags Added to Task Time Indices

eTable 11. Cognitive Control and State Engagement Variability During Drug Cue Paradigm (Cue Condition) With Lags Added to Task Time Indices

eTable 12. Associations Between Cognitive Control and Resting-State State Engagement Variability

© 2025 Ye J et al. *JAMA Network Open*.

eFigure 6. (A) State Engagement Variability and Stroop Interference Scores (B) Activations During the Drug Cue Task

eTable 13. Task Activation During the Cue Paradigm and Cognitive Control

eTable 14. Similarities Between HCP and CNP Brain States

eTable 15. State Engagement Variability During Naturalistic fMRI Using CNP Brain States (ANOVAs)

eFigure 7. Group Comparison Using State Engagement Variability Extracted Using CNP Brain States

eReferences.

This supplemental material has been provided by the authors to give readers additional information about their work.

© 2025 Ye J et al. *JAMA Network Open*.

eAppendix.

Functional magnetic resonance imaging (fMRI) acquisition and preprocessing

FMRI acquisition parameters were the same in both datasets and have been detailed in previous work (1,2). FMRI data were collected with harmonized Siemens 3T scanners using a 64-channel head coil at Yale's Magnetic Resonance Research Center. An anatomical scan was collected using a magnetization-prepared rapid gradient echo sequence (repetition time=2400ms, echo time=1.22ms, voxel size=1x1x1mm). FMRI was acquired using a multiband gradient echo-planar imaging sequence (repetition time=1000ms, echo time=30ms, voxel size=2x2x2mm, multiband factor=5).

Statistical Parametric Mapping (SPM12) performed slice time and motion correction for both datasets on the functional data. Additional data cleaning was carried out in BioImage Suite. Covariates of no interest were regressed, including linear and quadratic drift, white matter, cerebrospinal fluid, gray matter, and a 24-parameter motion model. The functional data were temporally smoothed (cutoff frequency around 0.12Hz).

Neuroimaging data were collected from 103 participants with OUD. Participants were recruited as a part of an NIH-funded study from Yale School of Medicine and the APT Foundation. All participants were stabilized on medications for opioid use disorder (MOUD). For the naturalistic paradigm, we first excluded participants who did not complete the task or had mean framewise displacement (MFD) over 0.2mm (N=24). One participant was excluded due to having epilepsy. After removing two additional participants with missing brain coverage, a final sample of 76 individuals with OUD was analyzed for group comparison. For the resting-state data, we removed 31 participants who did not complete the resting-state scan or showed MFD over 0.2mm. After excluding one participant with missing volume and one participant with missing brain coverage, we analyzed resting-state data from 70 participants with OUD. For the drug cue task, we removed 29 participants who did not complete the drug cue task or showed excessive motion (i.e., MFD > 0.2mm). Two participants were excluded due to missing time points in their scan. Two participants were removed due to missing brain coverage. After these exclusion criteria, we extracted brain dynamic measures from 70 participants with OUD.

The transdiagnostic study collected neuroimaging data from 307 participants. Out of these individuals, 294 adult participants completed the naturalistic paradigm. We excluded one participant with missing brain coverage. Three participants were further excluded due to having a wrong number of volumes. Of the 290 participants, 114 were considered healthy control (HC) based on our criteria (see **Methods**). We additionally removed five participants with MFD over 0.2mm. Nine repeated scans were excluded. Three more participants were removed due to issues with scanning sequences. A final sample of 97 HCs were included in the movie-watching analysis. The same transdiagnostic dataset also collected resting-state data from 107 participants who met criteria for HC. Five of these individuals were excluded for showing MFD over 0.2mm. Three more participants were removed due to scanning issues. Resting-state data from 99 HCs were analyzed.

© 2025 Ye J et al. *JAMA Network Open*.

Stroop-assessed cognitive control

As described above, fMRI data from 70 participants with OUD passed quality control. Three of these individuals did not have Stroop data and were excluded from further analysis related to cognitive control. During the Stroop task, participants were shown a word and were instructed to name the color of the word printed in. In the incongruent condition, participants responded to a color word printed in a conflicting color. In the control condition, participants were shown a rectangular block in one of the colors. We computed accuracy scores for the incongruent condition by calculating the portion of trials where participants responded correctly. Seven participants were excluded due to having outlier performance scores (determined using MATLAB; >3 median absolute deviations from the median; **eFigure 2**). We additionally extracted the average response time from all accurate trials in the incongruent condition. Two participants were removed due to missing data. We additionally excluded eight participants with outlier response time performance (same criteria as above). Stroop accuracy and response time did not differ significantly by sex (accuracy: $t(58)=-1.633$, $p=0.108$; response time: $t(55)=-0.272$, $p=0.787$).

Brain states identification

We replicated methods described in prior work to identify recurring brain states (3). Detailed descriptions can be found in Gao and colleagues (2023). In brief, nonlinear manifold learning and 2-step Diffusion Mapping projected task-based fMRI data from the Human Connectome Project into a low-dimensional space (3,4). We used minimally preprocessed HCP data from six different tasks (motor, working memory, social, emotional, relational, and gambling) from 390 participants (see 3 for information on quality control and exclusion criteria). Only participants who had available fMRI data from all fMRI conditions collected in the same task block order were included in this analysis.

After task data were projected to the low-dimensional space, time points showing similar activity patterns were located closer together. K-means clustering then identified four recurring brain states with distinct activation patterns. The number of brain states was determined using the Calinski-Harabasz criterion (5). We characterized these brain states as fixation, high-cognition, low-cognition, and transition based on the prominent task conditions associated with these brain states (**eTable 1**). For instance, the fixation state mainly included time points from the fixation condition. The high-cognition state included time points from complex cognitive paradigms such as working memory, emotion, relational, gambling, and social. The low-cognition state involved time points from the motor task, the 0-back working memory condition, and the neutral emotion condition. Lastly, the transition state consisted of time points from the cue condition across various task paradigms. The centroid of each state cluster was extracted to serve as a representative time point in later analyses.

In our prior work (6), we investigated how canonical brain networks contributed to these brain states. To this end, we identified the activated and deactivated brain regions (i.e., activation above or below 0, respectively; arbitrary unit) for each representative time point in a set of canonical brain

© 2025 Ye J et al. *JAMA Network Open*.

networks. The activation or deactivation percentage was next computed by dividing the number of activated or deactivated brain regions by the total number of brain regions in a network.

Different canonical networks were activated to varying extent in each brain state (**eTable 2**). However, brain network activation patterns largely followed what cognitive processes each brain state supported. For instance, the entire motor network was activated during low-cognition state whereas the high-cognition state was linked to frontoparietal network activation and default mode network deactivation.

© 2025 Ye J et al. *JAMA Network Open*.

Analysis approach

Since our framework outputs a SEV value for each brain state in each participant, Hotelling's T-square allowed us to include all SEV values in one multivariate model in order to assess group differences in SEV. This approach also gave us the opportunity to include other predictors (e.g., age and self-reported sex) in the model to explore their main and interaction effects. Leveraging multivariate analysis brings the advantages of limiting the number of statistical tests run as well as increasing power in detecting group differences. We checked that each brain state's SEV followed a Gaussian distribution (**eFigure 3**) before running the models.

Group comparison with resting-state fMRI

We performed supplementary analysis using resting-state fMRI data to examine whether altered SEV is a stable characteristic of OUD. Consistent with our main analysis, we observed a significant group ($F(4,161)=6.828$, $p<0.001$) and age ($F(4,161)=5.013$, $p<0.001$) main effect on SEV. There was no main sex ($F(1,161)=1.787$; $p=0.134$) or sex-by-group interaction ($F(4,161)=1.437$, $p=0.224$). Notably, there was a significant age-by-group interaction ($F(4,161)=2.442$, $p=0.049$) on resting-state SEV.

Follow-up ANOVAs were performed to investigate these effects in each brain state separately. Interestingly, group effect was no longer significant when each state was examined individually (**eTable 5**). This result suggests that multivariate analysis, such as T-square, may be more powerful at picking up on group differences. Additionally, while altered SEV may be observed in OUD in different paradigms, naturalistic stimuli may be better at capturing such differences than resting-state fMRI. We additionally observed significant age-by-group interaction for fixation, low-cognition and transition SEV (**eTable 5**). In line with our previous work (6), SEV for these three states decreased with age in HCs (**eFigure 4**). However, age-related decrease was not found in individuals with OUD (**eFigure 4**). As we also observed lower SEV in two of these states during movie-watching in individuals with OUD, one possibility is that SEV in OUD may be showing floor effects and has a more limited range to decrease. However, additional work is needed to test this hypothesis.

Relative state engagement (RSE) analysis

While the main interest of this study is to examine neural variability (i.e., SEV) in OUD, we also extracted and compared the amount of brain state engagement between groups. Specifically, we computed RSE, the sum of each state's engagement divided by the sum of engagement from all brain states included in the model. Since the sum of all RSE equals one, we examined each brain state's RSE separately. We performed ANOVAs to investigate group differences in RSE, including age and sex as covariates. Age-by-group and sex-by-group interactions were also explored.

We did not find significant group main effect on RSE during resting-state fMRI (fixation: $F(1,164)=2.911$, $p=0.090$; high-cognition: $F(1,164)=2.308$, $p=0.131$; low-cognition: $F(1,164)=3.431$, $p=0.066$; transition: $F(1,164)=1.678$, $p=0.197$; see other covariates in **eTable 6**).

© 2025 Ye J et al. *JAMA Network Open*.

However, during movie watching (**eTable 7**), we observed a significant main effect on high-cognition (F(1,166)=6.670, p=0.011) and low-cognition RSE (F(1,166)=6.634, p=0.011). Individuals with OUD engaged in the low-cognition state more while showing less high-cognition state recruitment (**eFigure 5**). There was no group main effect on fixation (F(1,166)=3.411, p=0.067) or transition RSE (F(1,166)=6.670, p=0.011). These findings demonstrate that during movie-watching, individuals with OUD engaged brain states differently than HCs, both in the variability and amount of engagement.

State engagement variability during rest condition and cognitive control assessed during Stroop incongruent condition

Recent literature suggests using a difference score to evaluate cognitive control may introduce concerns regarding test-retest reliability (7–10), convergent validity (11,12), and criterion validity (13,14). Work has additionally found that evaluating behavioral performance in one composite condition may better assess cognitive control (15,16). Given these concerns, we used accuracy (ACC) and accurate trial mean response time (RT) from the incongruent condition to assess cognitive control. However, we also computed the ACC (i.e., control condition ACC - incongruent condition ACC) as well as RT (i.e., incongruent accurate trial mean RT - control accurate trial mean RT) interference scores and evaluated their relationship with SEV. For ACC interference score analysis, we excluded five participants due to outlier performance scores. For RT interference score analysis, two participants were removed since response time information during the incongruent condition was missing. We additionally excluded four participants with outlier RT interference scores. ACC and RT interference scores did not differ significantly by sex (ACC interference: t(60)=0.976, p=0.333; RT interference: t(59)=-0.452, p=0.653). We found that lower SEV was associated with worse interference scores. Specifically, worse ACC and RT interference scores were linked to decreased transition (rho(60)=-0.41; p<0.01; q=0.02) and low-cognition SEV (rho(59)=-0.38; p<0.01; q=0.02) during the rest condition, respectively (**eFigure 6A; eTable 9**).

© 2025 Ye J et al. *JAMA Network Open*.

Control analysis to examine the association between cognitive control and SEV during resting-state fMRI

We performed a control analysis to investigate whether cognitive control was associated with SEV extracted during resting-state fMRI. Three participants were excluded for not having Stroop data. We also excluded participants with outlier or missing interference scores using the abovementioned criteria. Notably, cognitive control was unrelated to SEV during resting-state fMRI (**eTable 12**). These findings suggest that the rest period of the drug cue paradigm may serve as a particularly sensitive window into cognitive control.

General linear modeling (GLM)

As an exploratory analysis, we also examined whether brain responses during the drug cue paradigm were associated with cognitive control. For each participant, a GLM was used to model brain activity. The design matrix included the cue regressor, mean, linear, quadratic, and cubic trend terms, and a 24-parameter motion model. The cue regressor was convolved with a standard hemodynamic response function. Resultant beta maps were spatially smoothed with a 6-mm Gaussian kernel and warped into common space. Beta coefficients were extracted from each node of the Shen-268 atlas and correlated with ACC and RT interference scores. After correction for multiple comparisons, none of the brain nodes demonstrated responses significantly correlated with cognitive control (**eTable 13**). These results show that considering how brain responses change over time may provide additional information.

Validation analysis using brain states identified in clinical populations

In the current study, brain states were identified using the HCP dataset. This approach allowed us to identify brain states associated with various cognitive processes and prevent circular analysis. However, one potential concern is that since these brain states were identified in HCs, they might show a worse fit in individuals with OUD, confounding the group differences in SEV. Here, we performed validation analysis to investigate whether similar brain states can be identified in a clinical population and if we would observe similar SEV alterations in individuals with OUD using these brain states.

To identify brain states in a clinical population, we turned to the UCLA Consortium for Neuropsychiatric Phenomics (CNP) dataset (17). We used task-based fMRI data collected from individuals with schizophrenia, bipolar disorder (BD), and attention deficit hyperactivity disorder (ADHD). We chose these individuals so that the brain states were identified in neither HCs or individuals with OUD and thus the states would not be biased towards either of our study populations. Participants completed a series of fMRI tasks during their visit. This dataset was selected as each individual completed multiple fMRI paradigms, presenting the opportunity to identify different brain states. Time-locked tasks (i.e., participants were shown the same stimuli simultaneously) were selected for brain state identification. These included paired memory encoding, paired memory retrieval, spatial working memory, and task switching paradigms (see 17 for more information about these tasks).

FMRI data from the CNP dataset were preprocessed following a similar pipeline (3). We only included individuals who completed all four fMRI tasks (N=203), removed participants if any of their runs showed MFD over 0.2mm (N=24), and excluded one additional participant with missing brain coverage.

Out of the 178 participants who passed QC, 102 were patients (34 individuals with schizophrenia; 36 individuals with bipolar disorder; 32 individuals with ADHD). Following the same nonlinear manifold learning and 2-step Diffusion Mapping procedures as described above (3), we projected fMRI data from these individuals into a lower dimensional space. K-means clustering then identified three recurring brain states with distinct activation patterns. Consistent with our previous work, we used Calinski-Harabasz (5) to determine the number of brain states. Next, we identified the centroid of each cluster. Similarities between CNP and HCP brain states were examined using Pearson correlation. We found that brain states identified in a clinical population were significantly similar to those from HCP (**eTable 14**).

Next, we investigated whether the CNP brain states revealed similar SEV alterations in OUD. As we obtained more robust results using naturalistic fMRI, we repeated our analysis there using the CNP brain states. We first used a T-square test then ANOVAs to investigate group, sex, age, sex-by-group, and age-by-group effects.

We obtained remarkably similar results using the CNP brain states. Specifically, there was a significant group ($F(3,164)=6.511$; $p<0.001$), sex ($F(3,164)=3.689$; $p=0.013$), age main effect ($F(3,164)=13.915$; $p<0.001$) as well as a significant age-by-group interaction effect ($F(3,164)=3.366$; $p=0.020$). No sex-by-group interaction was observed ($F(3,164)=0.729$; $p=0.536$). ANOVAs to analyze each individual state also revealed that individuals with OUD showed lower SEV for two CNP brain states (**eTable 15; eFigure 7A**). While we found an age-related decrease in SEV in HCs, the same pattern was not seen in individuals with OUD (**eTable 15; eFigure 7B**). This is consistent with our findings in the resting-state fMRI analysis.

These findings provide preliminary evidence that similar brain states may be identified in individuals with psychiatric disorders and HCs. As the CNP brain states were identified in individuals with schizophrenia, BD, and ADHD, they are unlikely to bias one group over the other. Obtaining consistent results with the CNP brain states suggests that group differences may be potentially driven by how individuals engage brain states rather than the brain states themselves. However, future studies should test this hypothesis with more systematic analysis.

© 2025 Ye J et al. *JAMA Network Open*.

eFigure 1. Study Overview

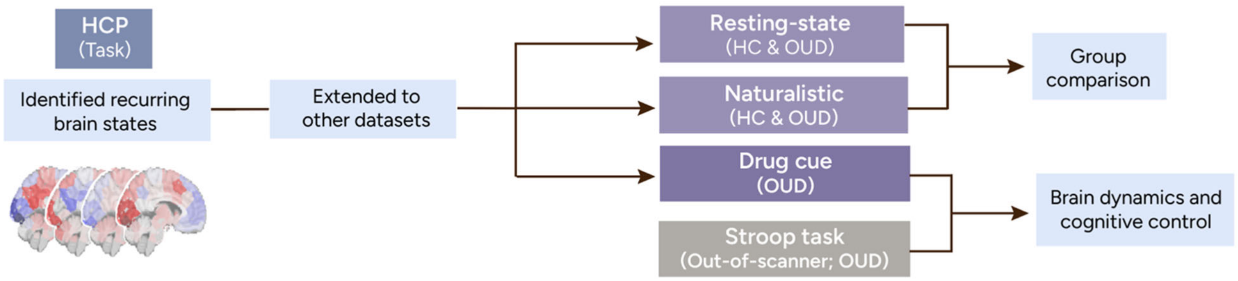

eFigure 1. We used multiple datasets in this study. First, recurring brain states are identified in the Human Connectome Project dataset. These brain states were then extended to naturalistic and resting-state fMRI data collected from both HCs and individuals with OUD as well as drug cue fMRI data from individuals with OUD. HC, healthy control; OUD, opioid use disorder.

© 2025 Ye J et al. *JAMA Network Open*.

eFigure 2. Distribution of Behavioral Performance During Stroop Incongruent Condition

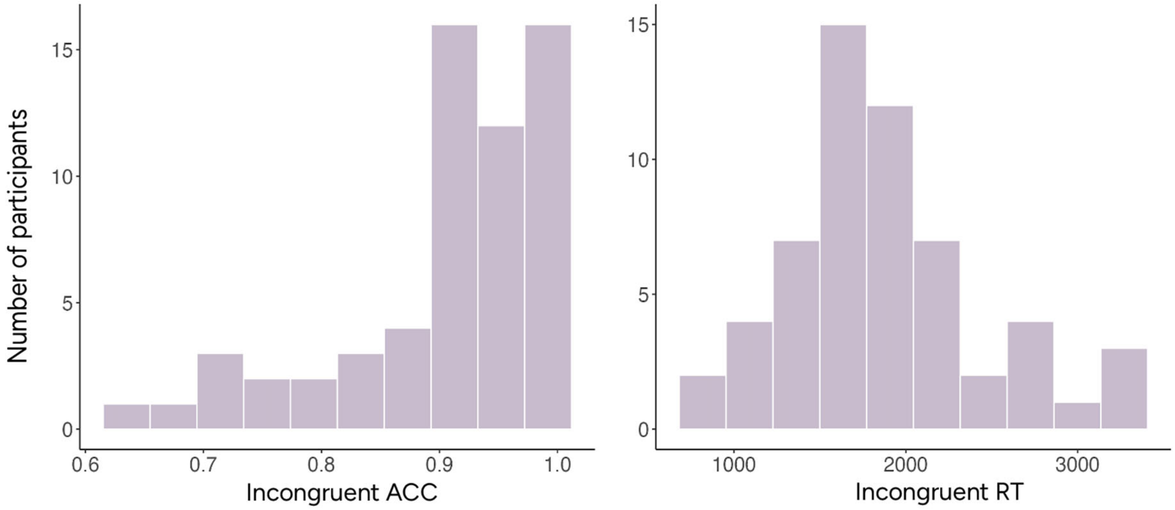

eFigure 2. Distribution of Behavioral Performance During Stroop Incongruent Condition

© 2025 Ye J et al. *JAMA Network Open*.

eTable 1. Number of Volumes Associated With Each Task Condition for the 4 Recurring Brain States

	Fixation	High-cognition	Low-cognition	Transition
Fixation	635	0	20	65
Cue	41	3	6	158
Working memory (0 back)	10	56	99	123
Working memory (2 back)	1	201	10	76
Emotion (Fear)	10	42	0	48
Emotion (Neutral)	23	12	99	16
Gambling (Win)	0	100	25	35
Gambling (Loss)	0	101	10	49
Motor (Tongue)	0	1	52	12
Motor (Left foot)	8	5	41	13
Motor (Left hand)	7	0	45	15
Motor (Right foot)	0	0	55	12
Motor (Right hand)	0	1	50	15
Social (Mental)	0	113	0	47
Social (Random)	0	111	0	109
Relational (Match)	3	9	17	40
Relational (Relation)	3	90	5	9

© 2025 Ye J et al. *JAMA Network Open*.

eTable 2. Networks Showing the Highest Activation and Deactivation Percentages for Each State

	Fixation	High-cognition	Low-cognition	Cue/transition
Activation percentages	DMN (88.89%)	VAs (100%)	Motor network (100%)	Visual I (100%)
	Motor network (85.71%)	Visual II (88.87%)	MF (86.21%)	VAs (72.22%)
	MF (82.76%)	FP (82.35%)	Cerebellum (84%)	Visual II (66.67%)
Deactivation percentages	VAs (94.44%)	Motor network (87.76%)	Visual I (100%)	MF (89.66%)
	Visual I (66.67%)	DMN (83.33%)	Visual II, VAs, and DMN (66.67%)	DMN (88.87%)
	Visual II (66.67%)	Subcortical (79.31%)		Motor (83.67%)

This table shows the canonical functional networks with the three highest activation and deactivation percentages for each brain state. The actual activation and deactivation percentage values were included in parentheses. DMN, default mode network; MF, medial frontal network; VAs, visual association network; FP, frontoparietal network.

© 2025 Ye J et al. *JAMA Network Open*.

eTable 3. Individuals With Opioid Use Disorder Who Reported Polysubstance Use

	Naturalistic (N=76)	Resting-state (N=71)	Drug cue (N=70)
Alcohol	59	57	57
Amphetamines	13	13	14
Barbiturates	5	5	4
Cannabis	69	64	61
Cocaine	67	64	63
Hallucinogens	31	31	29
Inhalants	6	5	6
Other sedatives/hypnotics/tranquilizers	20	20	21

© 2025 Ye J et al. *JAMA Network Open*.

eTable 4. State Engagement Variability During Naturalistic Stimuli ANOVA Covariates

	Sex	Age	Sex-by-group interaction	Age-by-group interaction
Fixation	F(1,166)=0.038; p=0.847	F(1,166)=4.484; p=0.036	F(1,166)=0.001; p=0.979	F(1,166)=5.593; p=0.019
High-cognition	F(1,166)=0.489; p=0.485	F(1,166)=7.488; p=0.007	F(1,166)=0.000; p=0.998	F(1,166)=4.400; p=0.037
Low-cognition	F(1,166)=1.109; p=0.294	F(1,166)=6.476; p=0.012	F(1,166)=0.377; p=0.540	F(1,166)=7.013; p=0.009
Transition	F(1,166)=0.133; p=0.716	F(1,166)=10.530; p=0.001	F(1,166)=0.168; p=0.682	F(1,166)=4.113; p=0.044

© 2025 Ye J et al. *JAMA Network Open*.

eFigure 3. Distribution of State Engagement Variability During Movie Watching (**A**) and Resting-State (**B**)

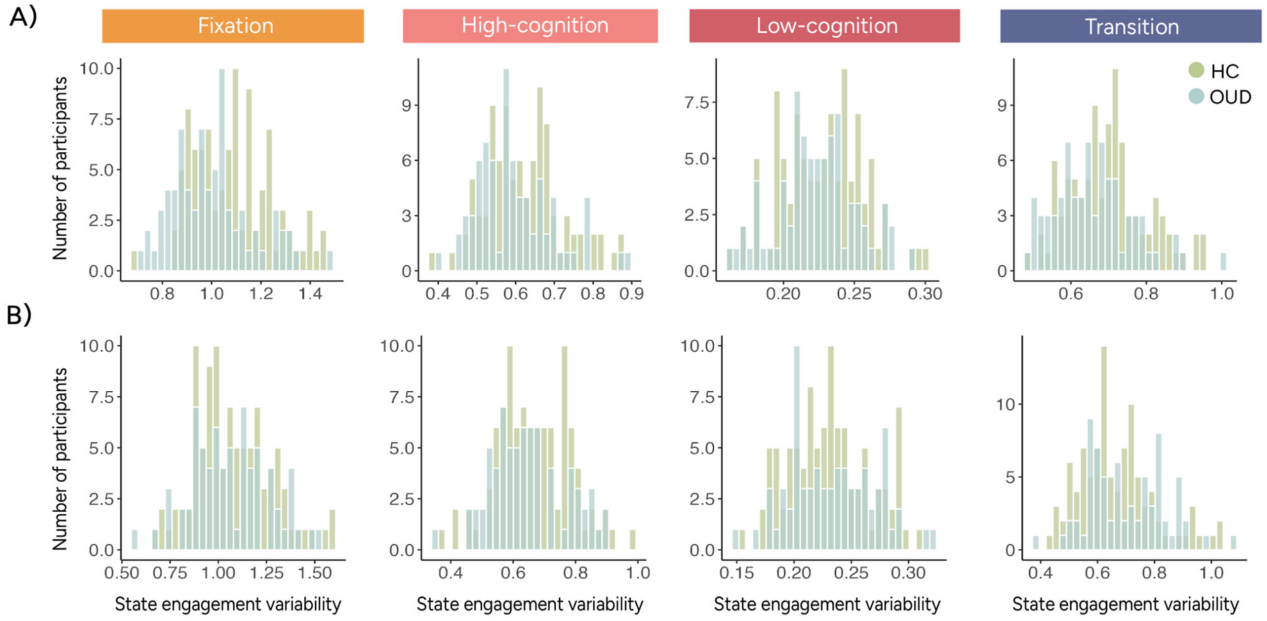

eFigure 3. Distribution of State Engagement Variability During Movie Watching (**A**) and Resting-State (**B**)

© 2025 Ye J et al. *JAMA Network Open*.

eTable 5. State Engagement Variability During Resting-State fMRI (ANOVAs)

	Group	Sex	Age	Sex-by-group interaction	Age-by-group interaction
Fixation	F(1,164)=0.128 p=0.721	F(1,164)=0.172 p=0.679	F(1,164)=9.404 p=0.003	F(1,164)=2.486 p=0.117	F(1,164)=4.620 p=0.033
High-cognition	F(1,164)=1.319 p=0.252	F(1,164)=1.268 p=0.262	F(1,164)=12.089 p=0.0006	F(1,164)=1.483 p=0.225	F(1,164)=3.413 p=0.066
Low-cognition	F(1,164)=0.188 p=0.665	F(1,164)=0.849 p=0.358	F(1,164)=10.942 p=0.001	F(1,164)=5.280 p=0.023	F(1,164)=9.504 p=0.002
Transition	F(1,164)=3.533 p=0.062	F(1,164)=0.016 p=0.900	F(1,164)=11.856 p=0.0007	F(1,164)=2.591 p=0.109	F(1,164)=4.581 p=0.034

© 2025 Ye J et al. *JAMA Network Open*.

eFigure 4. Age-by-Group Interaction in State Engagement Variability During Resting-State fMRI

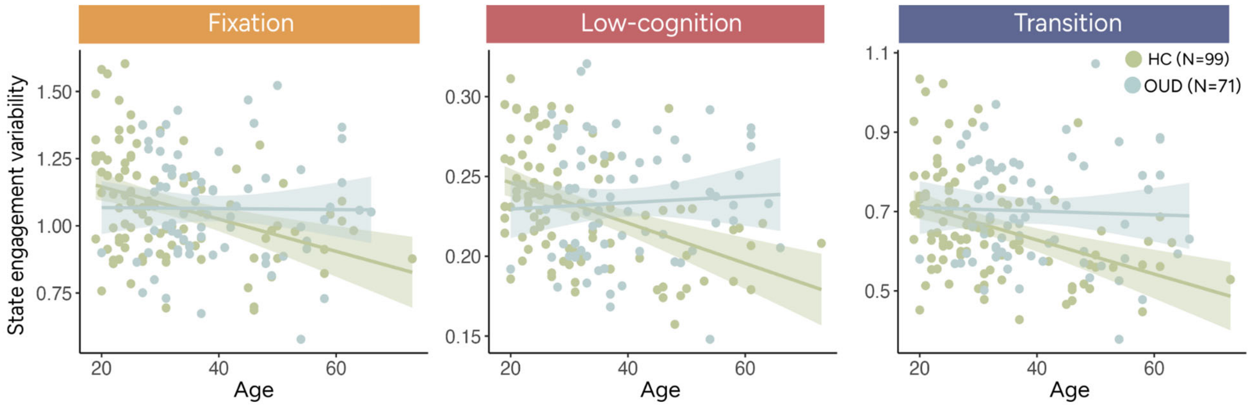

© 2025 Ye J et al. *JAMA Network Open*.

eFigure 5. Group Differences in Relative State Engagement During Movie-Watching

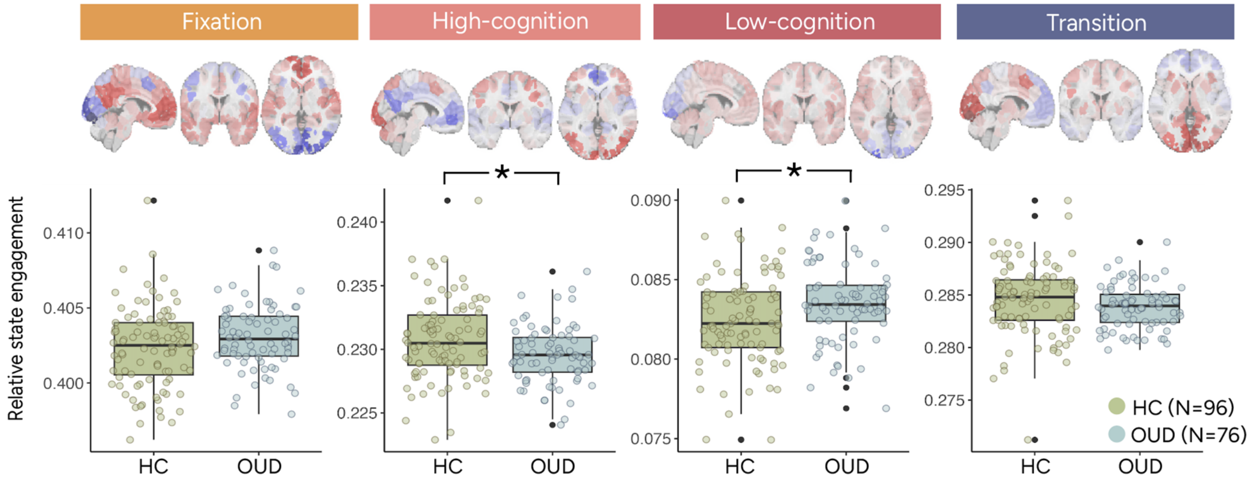

© 2025 Ye J et al. *JAMA Network Open*.

eTable 6. Relative State Engagement During Resting-State fMRI (ANOVAs)

	Sex	Age	Sex-by-group interaction	Age-by-group interaction
Fixation	F(1,164)=4.085; p=0.045	F(1,164)=4.314; p=0.039	F(1,164)=1.430; p=0.234	F(1,164)=0.625; p=0.430
High-cognition	F(1,164)=0.562; p=0.454	F(1,164)=0.113; p=0.737	F(1,164)=0.342; p=0.560	F(1,164)=0.324; p=0.570
Low-cognition	F(1,164)=2.721; p=0.101	F(1,164)=1.526; p=0.219	F(1,164)=1.666; p=0.199	F(1,164)=0.056; p=0.813
Transition	F(1,164)=1.038; p=0.310	F(1,164)=0.149; p=0.700	F(1,164)=0.180; p=0.672	F(1,164)=0.004; p=0.948

© 2025 Ye J et al. *JAMA Network Open*.

eTable 7. Relative State Engagement During Movie-Watching fMRI (ANOVAs)

	Sex	Age	Sex-by-group interaction	Age-by-group interaction
Fixation	F(1,166)=1.089; p=0.298	F(1,166)=0.014; p=0.906	F(1,166)=0.618; p=0.433	F(1,166)=0.084; p=0.773
High-cognition	F(1,166)=0.928; p=0.337	F(1,166)=0.389; p=0.534	F(1,166)=0.058; p=0.810	F(1,166)=0.068; p=0.794
Low-cognition	F(1,166)=0.250; p=0.617	F(1,166)=0.314; p=0.576	F(1,166)=0.825; p=0.365	F(1,166)=0.138; p=0.710
Transition	F(1,166)=1.785; p=0.183	F(1,166)=0.000; p=0.985	F(1,166)=0.004; p=0.952	F(1,166)=0.022; p=0.881

© 2025 Ye J et al. *JAMA Network Open*.

eTable 8. State Engagement Variability Between Rest and Cue Conditions

	Add 1 lag to time indices	Add 2 lags to time indices
Fixation	t(69)=2.591; p=0.012	t(69)=3.178; p=0.002
High-cognition	t(69)=2.702; p=0.009	t(69)=3.223; p=0.002
Low-cognition	t(69)=5.526; p<0.001	t(69)=6.482; p<0.001
Transition	t(69)=2.724; p=0.008	t(69)=3.423; p=0.001

© 2025 Ye J et al. *JAMA Network Open*.

eTable 9. State Engagement Variability During the Drug Cue Paradigm and Cognitive Control Assessed With Interference Scores

State engagement variability during cue and cognitive control						
	ACC Interference			RT Interference		
	rho	p	q	rho	p	q
Fixation	0.022	0.868	0.888	-0.100	0.443	0.885
High-cognition	0.023	0.858	0.888	-0.032	0.808	0.888
Low-cognition	0.030	0.820	0.888	-0.018	0.888	0.888
Transition	0.020	0.876	0.888	0.020	0.880	0.888
State engagement variability during rest and cognitive control						
	ACC Interference			RT Interference		
	rho	p	q	rho	p	q
Fixation	-0.302	0.017	0.091	-0.290	0.024	0.096
High-cognition	-0.247	0.053	0.170	0.182	0.160	0.366
Low-cognition	-0.088	0.498	0.885	-0.383	0.002	0.016
Transition	-0.405	0.001	0.016	-0.225	0.081	0.216

© 2025 Ye J et al. *JAMA Network Open*.

eFigure 6A. State Engagement Variability and Stroop Interference Scores

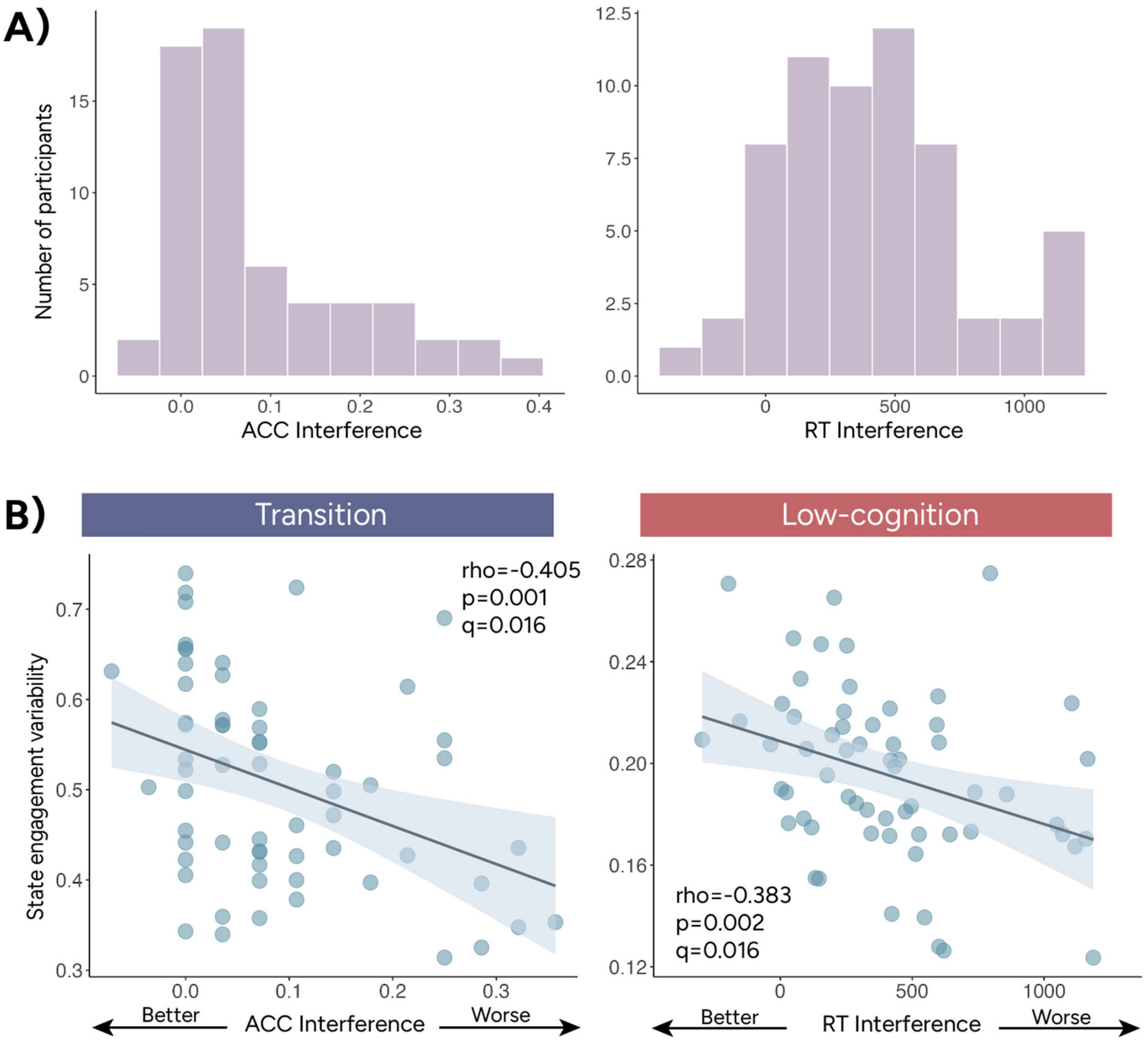

© 2025 Ye J et al. *JAMA Network Open*.

eTable 10. Cognitive Control and State Engagement Variability During Drug Cue Paradigm (Rest Condition) With Lags Added to Task Time Indices

	Add 1 lag to time indices		Add 2 lags to time indices	
	Incongruent ACC	Incongruent RT	Incongruent ACC	Incongruent RT
Fixation	rho=0.238; p=0.067	rho=-0.378; p=0.004	rho=0.211; p=0.105	rho=-0.326; p=0.014
High-cognition	rho=0.200; p=0.126	rho=-0.364; p=0.006	rho=0.204; p=0.118	rho=-0.303; p=0.022
Low-cognition	rho=0.106; p=0.422	rho=-0.105; p=0.434	rho=0.088; p=0.502	rho=-0.077; p=0.570
Transition	rho=0.320; p=0.013	rho=-0.320; p=0.016	rho=0.296; p=0.023	rho=-0.326; p=0.014

© 2025 Ye J et al. *JAMA Network Open*.

eTable 11. Cognitive Control and State Engagement Variability During Drug Cue Paradigm (Cue Condition) With Lags Added to Task Time Indices

	Add 1 lag to time indices		Add 2 lags to time indices	
	Incongruent ACC	Incongruent RT	Incongruent ACC	Incongruent RT
Fixation	rho=-0.008; p=0.953	rho=0.083; p=0.540	rho=0.046; p=0.728	rho=0.089; p=0.511
High-cognition	rho=0.008; p=0.952	rho=0.032; p=0.810	rho=0.025; p=0.848	rho=0.038; p=0.780
Low-cognition	rho=0.026; p=0.842	rho=0.017; p=0.897	rho=0.055; p=0.679	rho=-0.004; p=0.977
Transition	rho=0.059; p=0.657	rho=0.085; p=0.527	rho=0.082; p=0.536	rho=0.106; p=0.433

© 2025 Ye J et al. *JAMA Network Open*.

eTable 12. Associations Between Cognitive Control and Resting-State State Engagement Variability

	Accuracy Interference (N=60)	Response Time Interference (N=60’)
Fixation	rho=-0.074; p=0.575	rho=-0.051; p=0.700
High-cognition	rho=-0.015; p=0.911	rho=-0.130; p=0.322
Low-cognition	rho=-0.040; p=0.764	rho=-0.033; p=0.801
Transition	rho=-0.030; p=0.819	rho=-0.002; p=0.988

© 2025 Ye J et al. *JAMA Network Open*.

eFigure 6B. Activations During the Drug Cue Task

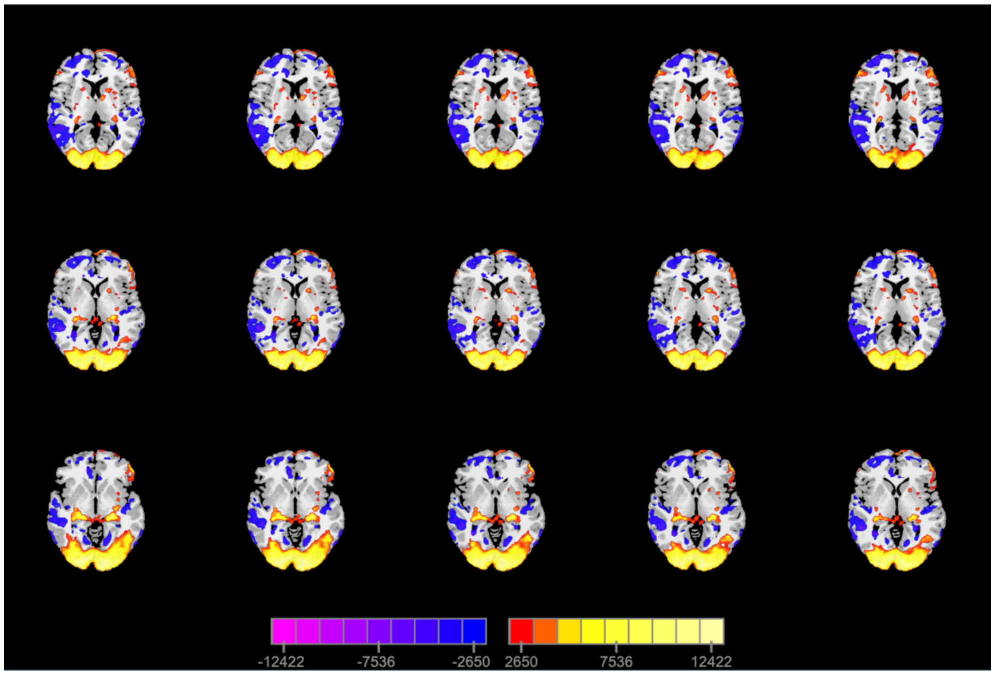

© 2025 Ye J et al. *JAMA Network Open*.

eTable 13. Task Activation During the Cue Paradigm and Cognitive Control

Node number	ACC Interference			RT Interference		
	r	p	q	r	p	q
1	0.13	0.33	0.89	0.19	0.14	0.99
2	0.05	0.70	0.89	0.18	0.16	0.99
3	0.04	0.75	0.89	0.13	0.34	0.99
4	-0.07	0.60	0.89	0.13	0.33	0.99
5	-0.09	0.49	0.89	0.05	0.73	0.99
6	-0.08	0.55	0.89	0.05	0.67	0.99
7	0.00	0.97	0.91	0.13	0.31	0.99
8	0.05	0.70	0.89	0.04	0.73	0.99
9	-0.15	0.23	0.89	-0.02	0.85	0.99
10	-0.09	0.47	0.89	-0.04	0.76	0.99
11	-0.20	0.11	0.89	0.10	0.46	0.99
12	-0.05	0.71	0.89	0.02	0.86	0.99
13	-0.21	0.10	0.89	-0.09	0.50	0.99
14	0.02	0.87	0.90	0.08	0.53	0.99
15	-0.18	0.16	0.89	0.00	0.98	1.00
16	0.05	0.69	0.89	-0.17	0.20	0.99
17	0.06	0.62	0.89	0.22	0.09	0.99
18	-0.01	0.91	0.90	0.11	0.38	0.99
19	0.04	0.74	0.89	0.06	0.67	0.99
20	-0.09	0.50	0.89	-0.05	0.70	0.99

© 2025 Ye J et al. *JAMA Network Open*.

Node number	ACC Interference			RT Interference		
	r	p	q	r	p	q
21	0.05	0.72	0.89	-0.02	0.85	0.99
22	0.21	0.10	0.89	0.01	0.95	1.00
23	0.04	0.75	0.89	-0.07	0.59	0.99
24	0.09	0.50	0.89	0.04	0.75	0.99
25	-0.01	0.93	0.90	-0.06	0.66	0.99
26	-0.05	0.70	0.89	0.17	0.18	0.99
27	0.09	0.48	0.89	-0.01	0.96	1.00
28	-0.15	0.25	0.89	0.08	0.55	0.99
29	0.00	0.99	0.91	0.22	0.10	0.99
30	-0.14	0.28	0.89	0.14	0.30	0.99
31	0.17	0.18	0.89	-0.04	0.74	0.99
32	-0.11	0.40	0.89	0.04	0.76	0.99
33	0.04	0.73	0.89	-0.06	0.64	0.99
34	-0.07	0.58	0.89	0.14	0.29	0.99
35	-0.14	0.27	0.89	0.06	0.66	0.99
36	-0.13	0.33	0.89	-0.04	0.76	0.99
37	0.00	0.98	0.91	-0.05	0.71	0.99
38	-0.19	0.13	0.89	0.03	0.81	0.99
39	0.04	0.78	0.89	-0.07	0.59	0.99
40	-0.05	0.72	0.89	-0.11	0.41	0.99
41	-0.06	0.63	0.89	0.09	0.48	0.99

© 2025 Ye J et al. *JAMA Network Open*.

Node number	ACC Interference			RT Interference		
	r	p	q	r	p	q
42	-0.19	0.13	0.89	0.04	0.77	0.99
43	-0.05	0.73	0.89	0.12	0.34	0.99
44	-0.05	0.69	0.89	0.18	0.16	0.99
45	-0.17	0.18	0.89	0.00	0.98	1.00
46	-0.13	0.32	0.89	-0.05	0.72	0.99
47	-0.16	0.21	0.89	0.20	0.12	0.99
48	0.09	0.51	0.89	0.07	0.58	0.99
49	0.04	0.79	0.89	0.13	0.30	0.99
50	-0.02	0.90	0.90	-0.06	0.62	0.99
51	0.02	0.88	0.90	0.00	0.99	1.00
52	0.00	0.97	0.91	0.06	0.63	0.99
53	0.03	0.82	0.89	-0.06	0.67	0.99
54	0.00	0.99	0.91	-0.21	0.11	0.99
55	0.09	0.48	0.89	0.12	0.36	0.99
56	-0.05	0.72	0.89	0.10	0.44	0.99
57	-0.08	0.51	0.89	0.04	0.74	0.99
58	-0.12	0.35	0.89	-0.02	0.85	0.99
59	-0.05	0.70	0.89	-0.03	0.84	0.99
60	-0.12	0.35	0.89	-0.04	0.76	0.99
61	0.03	0.80	0.89	-0.04	0.78	0.99
62	-0.05	0.68	0.89	-0.15	0.24	0.99

© 2025 Ye J et al. *JAMA Network Open*.

Node number	ACC Interference			RT Interference		
	r	p	q	r	p	q
63	-0.04	0.76	0.89	-0.10	0.46	0.99
64	-0.09	0.51	0.89	-0.10	0.45	0.99
65	0.05	0.71	0.89	-0.14	0.30	0.99
66	0.03	0.79	0.89	-0.02	0.86	0.99
67	0.22	0.09	0.89	-0.04	0.75	0.99
68	-0.16	0.20	0.89	-0.13	0.32	0.99
69	0.15	0.24	0.89	0.00	0.99	1.00
70	0.06	0.62	0.89	0.03	0.82	0.99
71	0.02	0.90	0.90	-0.03	0.84	0.99
72	0.05	0.70	0.89	0.02	0.86	0.99
73	0.06	0.63	0.89	-0.01	0.92	1.00
74	-0.01	0.92	0.90	-0.12	0.35	0.99
75	0.00	0.99	0.91	0.12	0.37	0.99
76	0.13	0.32	0.89	-0.08	0.55	0.99
77	-0.12	0.34	0.89	-0.04	0.77	0.99
78	0.28	0.03	0.89	0.03	0.83	0.99
79	0.14	0.27	0.89	0.02	0.85	0.99
80	0.02	0.86	0.90	0.14	0.29	0.99
81	0.18	0.17	0.89	0.12	0.37	0.99
82	0.05	0.68	0.89	0.00	0.99	1.00
83	-0.17	0.19	0.89	-0.01	0.93	1.00

© 2025 Ye J et al. *JAMA Network Open*.

Node number	ACC Interference			RT Interference		
	r	p	q	r	p	q
84	-0.14	0.27	0.89	0.00	0.99	1.00
85	-0.04	0.79	0.89	0.06	0.65	0.99
86	-0.13	0.30	0.89	-0.08	0.55	0.99
87	-0.05	0.71	0.89	-0.08	0.54	0.99
88	-0.13	0.30	0.89	0.07	0.58	0.99
89	-0.12	0.34	0.89	-0.12	0.36	0.99
90	-0.15	0.24	0.89	0.06	0.67	0.99
91	-0.15	0.25	0.89	0.14	0.28	0.99
92	-0.06	0.67	0.89	0.18	0.16	0.99
93	-0.30	0.02	0.89	0.00	1.00	1.00
94	0.03	0.81	0.89	0.17	0.19	0.99
95	-0.24	0.06	0.89	-0.01	0.95	1.00
96	-0.12	0.34	0.89	-0.10	0.43	0.99
97	-0.04	0.78	0.89	0.14	0.27	0.99
98	-0.08	0.56	0.89	0.00	0.98	1.00
99	-0.01	0.96	0.91	0.14	0.28	0.99
100	0.07	0.61	0.89	0.03	0.83	0.99
101	-0.26	0.04	0.89	0.09	0.51	0.99
102	0.05	0.72	0.89	-0.03	0.85	0.99
103	-0.19	0.15	0.89	0.02	0.91	1.00
104	-0.03	0.79	0.89	0.07	0.60	0.99

© 2025 Ye J et al. *JAMA Network Open*.

Node number	ACC Interference			RT Interference		
	r	p	q	r	p	q
105	-0.12	0.37	0.89	0.10	0.46	0.99
106	-0.08	0.54	0.89	0.08	0.52	0.99
107	-0.04	0.76	0.89	0.31	0.02	0.99
108	-0.10	0.44	0.89	0.10	0.46	0.99
109	-0.11	0.37	0.89	0.02	0.91	1.00
110	-0.17	0.19	0.89	0.02	0.90	1.00
111	0.05	0.69	0.89	0.09	0.50	0.99
112	-0.03	0.82	0.89	0.09	0.51	0.99
113	-0.13	0.31	0.89	0.01	0.92	1.00
114	0.05	0.70	0.89	0.11	0.41	0.99
115	-0.11	0.40	0.89	0.10	0.46	0.99
116	-0.12	0.34	0.89	0.06	0.63	0.99
117	-0.20	0.13	0.89	-0.01	0.93	1.00
118	-0.10	0.43	0.89	0.21	0.11	0.99
119	-0.08	0.52	0.89	0.07	0.61	0.99
120	-0.01	0.95	0.91	0.03	0.83	0.99
121	-0.04	0.73	0.89	0.00	0.98	1.00
122	-0.11	0.41	0.89	0.00	1.00	1.00
123	0.04	0.77	0.89	0.16	0.22	0.99
124	-0.05	0.71	0.89	-0.14	0.27	0.99
125	-0.04	0.76	0.89	0.08	0.55	0.99

© 2025 Ye J et al. *JAMA Network Open*.

Node number	ACC Interference			RT Interference		
	r	p	q	r	p	q
126	-0.13	0.30	0.89	0.13	0.32	0.99
127	-0.04	0.77	0.89	0.14	0.27	0.99
128	-0.06	0.63	0.89	0.07	0.57	0.99
129	0.10	0.44	0.89	0.19	0.15	0.99
130	0.08	0.53	0.89	0.08	0.56	0.99
131	-0.12	0.34	0.89	-0.08	0.53	0.99
132	-0.24	0.06	0.89	0.02	0.88	1.00
133	-0.17	0.19	0.89	-0.05	0.69	0.99
134	-0.01	0.91	0.90	0.14	0.29	0.99
135	-0.03	0.83	0.90	0.04	0.77	0.99
136	0.23	0.07	0.89	-0.07	0.59	0.99
137	0.05	0.69	0.89	-0.08	0.56	0.99
138	-0.05	0.68	0.89	0.02	0.88	0.99
139	0.05	0.69	0.89	-0.04	0.77	0.99
140	-0.07	0.57	0.89	-0.01	0.92	1.00
141	-0.12	0.34	0.89	-0.03	0.83	0.99
142	-0.09	0.49	0.89	0.01	0.94	1.00
143	0.06	0.62	0.89	-0.04	0.76	0.99
144	-0.13	0.33	0.89	0.06	0.63	0.99
145	-0.18	0.16	0.89	-0.10	0.43	0.99
146	-0.02	0.89	0.90	-0.05	0.70	0.99

© 2025 Ye J et al. *JAMA Network Open*.

Node number	ACC Interference			RT Interference		
	r	p	q	r	p	q
147	0.11	0.38	0.89	-0.09	0.50	0.99
148	-0.10	0.46	0.89	-0.19	0.15	0.99
149	-0.06	0.62	0.89	-0.11	0.41	0.99
150	-0.11	0.38	0.89	0.04	0.78	0.99
151	0.14	0.27	0.89	-0.08	0.54	0.99
152	0.04	0.77	0.89	0.11	0.41	0.99
153	-0.04	0.76	0.89	0.06	0.62	0.99
154	0.14	0.27	0.89	0.15	0.23	0.99
155	-0.09	0.48	0.89	-0.01	0.94	1.00
156	0.03	0.84	0.90	-0.01	0.96	1.00
157	0.14	0.29	0.89	-0.04	0.78	0.99
158	0.10	0.45	0.89	-0.05	0.68	0.99
159	0.07	0.61	0.89	-0.04	0.79	0.99
160	-0.08	0.54	0.89	0.09	0.51	0.99
161	-0.17	0.18	0.89	-0.05	0.68	0.99
162	-0.11	0.38	0.89	0.05	0.71	0.99
163	0.03	0.82	0.89	0.08	0.54	0.99
164	-0.13	0.30	0.89	0.11	0.39	0.99
165	0.06	0.67	0.89	-0.02	0.86	0.99
166	-0.05	0.67	0.89	-0.04	0.74	0.99
167	-0.02	0.88	0.90	0.00	1.00	1.00

© 2025 Ye J et al. *JAMA Network Open*.

Node number	ACC Interference			RT Interference		
	r	p	q	r	p	q
168	-0.03	0.80	0.89	0.13	0.34	0.99
169	-0.01	0.92	0.90	0.07	0.57	0.99
170	-0.05	0.71	0.89	-0.21	0.10	0.99
171	0.02	0.87	0.90	-0.04	0.78	0.99
172	-0.06	0.62	0.89	-0.04	0.77	0.99
173	-0.05	0.72	0.89	-0.20	0.13	0.99
174	0.00	1.00	0.91	0.05	0.70	0.99
175	-0.01	0.93	0.90	0.07	0.61	0.99
176	-0.20	0.12	0.89	-0.03	0.83	0.99
177	0.11	0.39	0.89	-0.07	0.60	0.99
178	0.00	1.00	0.91	0.20	0.12	0.99
179	-0.03	0.85	0.90	0.02	0.87	0.99
180	-0.11	0.40	0.89	-0.09	0.49	0.99
181	-0.21	0.10	0.89	-0.04	0.77	0.99
182	-0.06	0.66	0.89	-0.03	0.82	0.99
183	-0.02	0.91	0.90	-0.16	0.22	0.99
184	-0.08	0.55	0.89	0.17	0.20	0.99
185	-0.02	0.90	0.90	-0.18	0.16	0.99
186	-0.04	0.74	0.89	-0.04	0.76	0.99
187	-0.07	0.59	0.89	0.02	0.85	0.99
188	0.00	0.99	0.91	0.01	0.96	1.00

© 2025 Ye J et al. *JAMA Network Open*.

Node number	ACC Interference			RT Interference		
	r	p	q	r	p	q
189	0.08	0.56	0.89	0.11	0.38	0.99
190	-0.02	0.90	0.90	-0.05	0.69	0.99
191	0.00	0.99	0.91	-0.08	0.52	0.99
192	0.04	0.73	0.89	-0.17	0.19	0.99
193	0.02	0.87	0.90	0.03	0.83	0.99
194	-0.17	0.18	0.89	-0.01	0.91	1.00
195	-0.15	0.26	0.89	0.03	0.79	0.99
196	-0.20	0.11	0.89	0.06	0.62	0.99
197	-0.08	0.53	0.89	-0.04	0.75	0.99
198	0.09	0.48	0.89	-0.04	0.74	0.99
199	0.04	0.75	0.89	0.07	0.61	0.99
200	0.09	0.49	0.89	-0.04	0.73	0.99
201	-0.03	0.80	0.89	0.01	0.95	1.00
202	0.01	0.92	0.90	-0.06	0.63	0.99
203	0.03	0.80	0.89	-0.15	0.25	0.99
204	0.03	0.81	0.89	-0.05	0.72	0.99
205	-0.09	0.49	0.89	-0.09	0.49	0.99
206	0.14	0.28	0.89	-0.02	0.89	1.00
207	0.09	0.46	0.89	0.03	0.85	0.99
208	0.02	0.90	0.90	0.11	0.38	0.99
209	0.04	0.75	0.89	-0.16	0.21	0.99

© 2025 Ye J et al. *JAMA Network Open*.

Node number	ACC Interference			RT Interference		
	r	p	q	r	p	q
210	0.10	0.45	0.89	-0.15	0.24	0.99
211	0.13	0.33	0.89	-0.02	0.85	0.99
212	0.27	0.03	0.89	0.09	0.48	0.99
213	0.02	0.89	0.90	0.03	0.80	0.99
214	0.11	0.42	0.89	0.05	0.68	0.99
215	0.07	0.57	0.89	-0.05	0.72	0.99
216	-0.07	0.60	0.89	-0.04	0.79	0.99
217	-0.21	0.10	0.89	0.08	0.53	0.99
218	-0.18	0.15	0.89	-0.10	0.43	0.99
219	-0.19	0.14	0.89	-0.04	0.76	0.99
220	-0.15	0.24	0.89	-0.11	0.38	0.99
221	-0.17	0.19	0.89	0.04	0.78	0.99
222	-0.07	0.61	0.89	-0.22	0.09	0.99
223	-0.07	0.58	0.89	0.03	0.82	0.99
224	-0.21	0.09	0.89	-0.04	0.77	0.99
225	-0.13	0.31	0.89	0.03	0.82	0.99
226	-0.19	0.15	0.89	0.12	0.36	0.99
227	-0.09	0.48	0.89	-0.05	0.72	0.99
228	0.00	0.98	0.91	0.17	0.20	0.99
229	0.07	0.62	0.89	0.03	0.80	0.99
230	-0.01	0.91	0.90	0.08	0.55	0.99

© 2025 Ye J et al. *JAMA Network Open*.

Node number	ACC Interference			RT Interference		
	r	p	q	r	p	q
231	-0.04	0.77	0.89	0.21	0.10	0.99
232	0.22	0.09	0.89	0.04	0.75	0.99
233	0.07	0.58	0.89	0.09	0.52	0.99
234	-0.02	0.89	0.90	-0.16	0.23	0.99
235	-0.10	0.46	0.89	0.10	0.46	0.99
236	-0.07	0.58	0.89	0.13	0.32	0.99
237	-0.15	0.23	0.89	0.03	0.80	0.99
238	-0.06	0.66	0.89	0.10	0.45	0.99
239	-0.22	0.09	0.89	0.17	0.20	0.99
240	-0.07	0.56	0.89	0.10	0.43	0.99
241	0.08	0.56	0.89	0.24	0.07	0.99
242	-0.01	0.94	0.91	0.09	0.47	0.99
243	-0.01	0.95	0.91	0.05	0.70	0.99
244	-0.16	0.22	0.89	0.16	0.22	0.99
245	-0.18	0.16	0.89	0.02	0.86	0.99
246	0.01	0.92	0.90	0.15	0.24	0.99
247	-0.07	0.59	0.89	0.08	0.53	0.99
248	-0.04	0.76	0.89	0.09	0.51	0.99
249	-0.03	0.82	0.89	0.15	0.26	0.99
250	-0.17	0.18	0.89	-0.02	0.85	0.99
251	-0.06	0.67	0.89	0.18	0.16	0.99

© 2025 Ye J et al. *JAMA Network Open*.

Node number	ACC Interference			RT Interference		
	r	p	q	r	p	q
252	0.05	0.70	0.89	0.28	0.03	0.99
253	0.04	0.75	0.89	0.09	0.47	0.99
254	-0.09	0.47	0.89	0.08	0.56	0.99
255	-0.25	0.05	0.89	0.00	0.99	1.00
256	0.04	0.77	0.89	0.07	0.57	0.99
257	-0.03	0.81	0.89	0.24	0.06	0.99
258	0.10	0.42	0.89	0.24	0.06	0.99
259	-0.08	0.53	0.89	0.11	0.38	0.99
260	-0.03	0.84	0.90	0.04	0.79	0.99
261	0.03	0.79	0.89	-0.12	0.34	0.99
262	0.11	0.39	0.89	0.05	0.68	0.99
263	-0.11	0.41	0.89	0.05	0.69	0.99
264	-0.05	0.72	0.89	0.12	0.37	0.99
265	-0.29	0.02	0.89	0.01	0.92	1.00
266	0.04	0.75	0.89	0.13	0.34	0.99
267	-0.18	0.16	0.89	-0.07	0.60	0.99
268	0.08	0.54	0.89	0.09	0.51	0.99

© 2025 Ye J et al. *JAMA Network Open*.

eTable 14. Similarities Between HCP and CNP Brain States

	Fixation	High-cognition	Low-cognition	Transition
CNP State 1	r=-0.856; p<0.001	r=0.652; p<0.001	r=-0.653; p<0.001	r=0.870; p<0.001
CNP State 2	r=0.856; p<0.001	r=-0.646; p<0.001	r=0.626; p<0.001	r=-0.864; p<0.001
CNP State 3	r=-0.680; p<0.001	r=0.569; p<0.001	r=-0.128; p=0.040	r=0.545; p<0.001

© 2025 Ye J et al. *JAMA Network Open*.

eTable 15. State Engagement Variability During Naturalistic fMRI Using CNP Brain States (ANOVAs)

	Group	Sex	Age	Sex-by-group interaction	Age-by-group interaction
CNP state 1	F(1,166)=4.546 p=0.035	F(1,166)=0.690 p=0.407	F(1,166)=32.659 p<0.001	F(1,166)=0.016 p=0.900	F(1,166)=9.278 p=0.003
CNP state 2	F(1,166)=10.710 p=0.001	F(1,166)=1.196 p=0.276	F(1,166)=27.839 p<0.001	F(1,166)=0.005 p=0.942	F(1,166)=10.185 p=0.002
CNP state 2	F(1,166)=0.031 p=0.859	F(1,166)=4.172 p=0.043	F(1,166)=33.790 p<0.001	F(1,166)=1.139 p=0.287	F(1,166)=3.468 p=0.064

© 2025 Ye J et al. *JAMA Network Open*.

eFigure 7. Group Comparison Using State Engagement Variability Extracted Using CNP Brain States

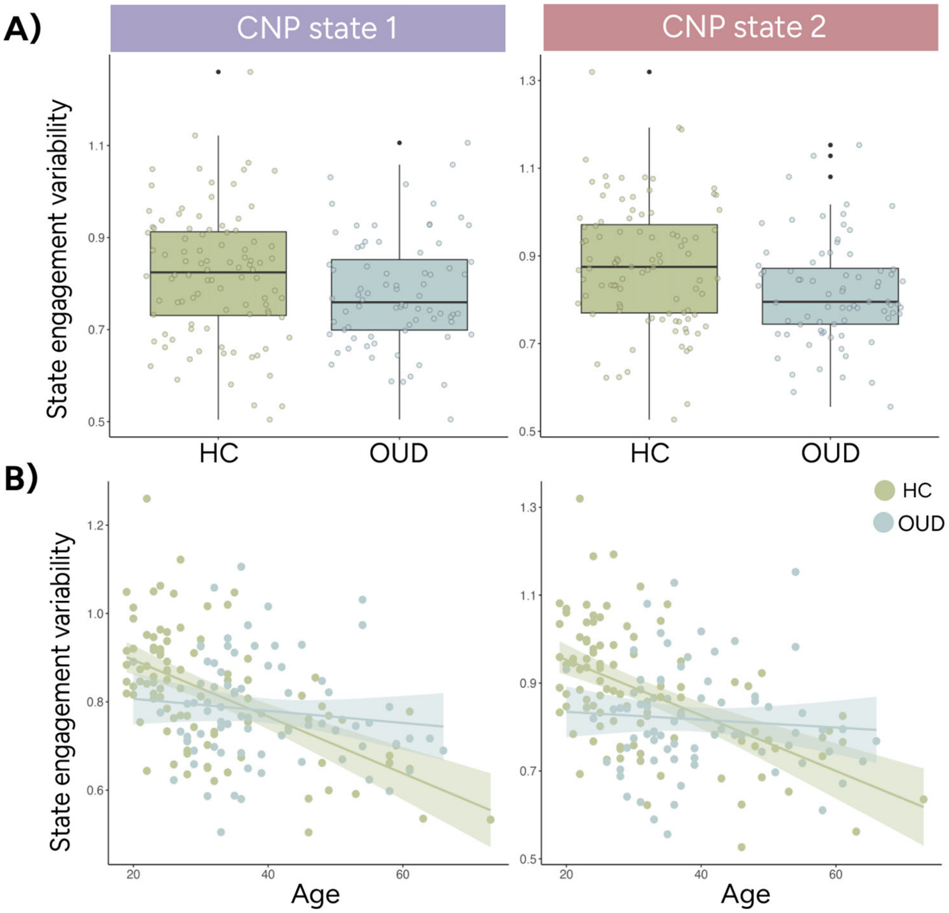

© 2025 Ye J et al. *JAMA Network Open*.

eReferences.

1. Greene, A. S. *et al.* Brain–phenotype models fail for individuals who defy sample stereotypes. *Nature* **609**, 109–118 (2022).
2. Mehta, S. *et al.* Alterations in Volume and Intrinsic Resting-state Functional Connectivity in Individuals with Opioid Use Disorder. *Radiology* (in press)
3. Gao, S., Mishne, G. & Scheinost, D. Nonlinear manifold learning in functional magnetic resonance imaging uncovers a low-dimensional space of brain dynamics. *Hum. Brain Mapp.* **42**, 4510–4524 (2021).
4. Van Essen, D. C. *et al.* The WU-Minn Human Connectome Project: An overview. *NeuroImage* **80**, 62–79 (2013).
5. Caliński, T. & Harabasz, J. A dendrite method for cluster analysis. *Commun. Stat.* **3**, 1–27 (1974).
6. Ye, J. *et al.* Altered Brain Dynamics Across Bipolar Disorder and Schizophrenia During Rest and Task Switching Revealed by Overlapping Brain States. *Biol. Psychiatry* **94**, 580–590 (2023).
7. Hedge, C., Powell, G. & Sumner, P. The reliability paradox: Why robust cognitive tasks do not produce reliable individual differences. *Behav. Res. Methods* **50**, 1166–1186 (2018).
8. Enkavi, A. Z. *et al.* Large-scale analysis of test–retest reliabilities of self-regulation measures. *Proc. Natl. Acad. Sci.* **116**, 5472–5477 (2019).
9. Draheim, C., Mashburn, C. A., Martin, J. D. & Engle, R. W. Reaction time in differential and developmental research: A review and commentary on the problems and alternatives. *Psychol. Bull.* **145**, 508–535 (2019).
10. Rouder, J. N., Kumar, A. & Haaf, J. M. Why many studies of individual differences with inhibition tasks may not localize correlations. *Psychon. Bull. Rev.* **30**, 2049–2066 (2023).
11. Rey-Mermet, A., Gade, M. & Oberauer, K. Should we stop thinking about inhibition? Searching for individual and age differences in inhibition ability. *J. Exp. Psychol. Learn. Mem. Cogn.* **44**, 501–526 (2018).
12. Hedge, C., Powell, G., Bompas, A. & Sumner, P. Strategy and processing speed eclipse individual differences in control ability in conflict tasks. *J. Exp. Psychol. Learn. Mem. Cogn.* **48**, 1448–1469 (2022).
13. Eisenberg, I. W. *et al.* Uncovering the structure of self-regulation through data-driven ontology discovery. *Nat. Commun.* **10**, 2319 (2019).
14. Saunders, B., Milyavskaya, M., Etz, A., Randles, D. & Inzlicht, M. Reported Self-control is not Meaningfully Associated with Inhibition-related Executive Function: A Bayesian Analysis. *Collabra Psychol.* **4**, 39 (2018).
15. Löffler, C., Frischkorn, G. T., Hagemann, D., Sadus, K. & Schubert, A.-L. The common factor of executive functions measures nothing but speed of information uptake. *Psychol. Res.* **88**, 1092–1114 (2024).
16. Weigard, A., Clark, D. A. & Sripada, C. Cognitive efficiency beats top-down control as a reliable individual difference dimension relevant to self-control. *Cognition* **215**, 104818 (2021).
17. Poldrack, R. A. *et al.* A phenome-wide examination of neural and cognitive function. *Sci. Data* **3**, 160110 (2016).

© 2025 Ye J et al. *JAMA Network Open*.
