# Supplementary material for: Neural Variability and Cognitive Control in Individuals With Opioid Use Disorder
Source: JAMA Netw Open. 2025 Jan 17;8(1):e2455165. doi: 10.1001/jamanetworkopen.2024.55165 (PMC11742521; doi:10.1001/jamanetworkopen.2024.55165)
Supplement: Supplement 2. — Data Sharing Statement [file jamanetwopen-e2455165-s002.pdf]

Data Sharing Statement

Ye. Neural Variability and Cognitive Control in Individuals With Opioid Use Disorder. *JAMA Netw Open*. Published January 17, 2025. doi:10.1001/jamanetworkopen.2024.55165

Data
Data available: No
